# A review of pharmaceutical and personal care products and atopic dermatitis based on epidemiological and basic research findings

**DOI:** 10.3389/fpubh.2025.1642873

**Published:** 2025-09-25

**Authors:** Shushu Xie, Yan Jiang, Zhiqin Hu, Zhishan Ding, Jie Yu, Xiaoqing Ye

**Affiliations:** ^1^School of Medical Technology and Information Engineering, Zhejiang Chinese Medical University, Hangzhou, China; ^2^Puer Kunhong Biotechnology Company, Pu'er, China

**Keywords:** pharmaceutical and personal care products, emerging contaminants, atopic dermatitis, immunotoxicity, risk factors, health hazards

## Abstract

Pharmaceutical and personal care products (PPCPs) are emerging pollutants that have been found to be related to allergic diseases. Currently, a large amount of research focused on the association between PPCPs and atopic dermatitis (AD), but there has been no evaluation of existing evidence on this topic. Here, we reviewed epidemiological and toxicological studies from 2014 to 2024, with a focus on common PPCPs such as antibiotics, phthalates, p-hydroxybenzoic acid, etc. We found that most PPCPs are positively correlated with the onset of AD, with particular attention paid to exposure during pregnancy and infancy. This reminds AD patients to be cautious when taking medication and choosing nursing products. Animal studies have shown that the pathogenesis of PPCPs may be mediated by dysbiosis, immune imbalance and oxidative stress. Despite inconsistent results in existing research, PPCPs are confirmed to be unfavorable drivers of AD occurrence and progression. Clarifying their potential link with AD is critical for informing subsequent policy and regulatory decisions.

## Graphical abstract

This image is created using BY Figdraw.
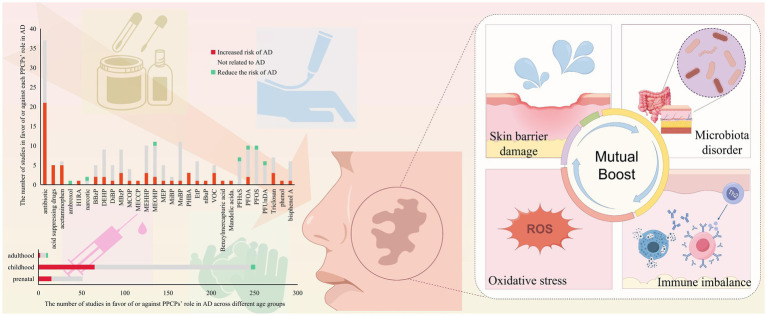

## 1 Introduction

Atopic dermatitis (AD) is a chronic disease affecting up to 20% of children and 3% of adults worldwide (1). Women are more susceptible to AD with approximately 13 million more women than men affected globally (2). The intense and persistent itching experienced by patients with AD can impose a significant burden on families, both financially and emotionally, making it a social problem. A growing number of studies have concluded that AD is the result of a complex interaction between genetic and environmental factors (3, 4). The potential association between environmental pollution and AD has gained significant attention in recent discussions (5).

Pharmaceutical and personal care products (PPCPs) are classified as “pseudo-persistent” pollutants because they are continuously introduced into the environment and remain long-lasting in aquatic environments (6). Through the food chain, PPCPs accumulate in the human body and have been widely detected (7, 8). Although most PPCPs entering the body are metabolized by the kidneys (9), studies have reported the detection of PPCPs residues in the blood, organs, and even hypothalamic tissues (10, 11). To date, PPCPs have been found to interfere with human microbiota and are associated with a variety of diseases (12, 13).

Patients with AD are more susceptible to PPCPs because of their prolonged use of emollient products and a long course of medications (14). Mounting evidence suggests that PPCPs exposure, particularly during in uterus and early life stages, is strongly associated with the development of allergic diseases, including AD. To evaluate the association between PPCPs and AD, we conducted a systematic review of epidemiologic studies from 2014 to 2024, aiming to provide evidence elucidating the pathogenic risk of PPCPs. Due to the fact that the majority of epidemiological study findings center on pediatrics, although we did not subjectively screen literature, our review inevitably focused on the harm caused to pediatrics by early exposure to PPCPs. As for research findings related to adults, we have not excluded them but have not highlighted them either.

## 2 Retrieval strategy

We searched the PubMed and CNKI databases for studies related to PPCPs and AD from 2014 to 2024. The search keywords included “atopic dermatitis” “atopic eczema” “expose” “exposure” “pollutant” “environmental pollution” “PPCPs” “care products” “detergent” “hair dye” “nail polish” and all pollutants included in the following text.

**Figure 1:**
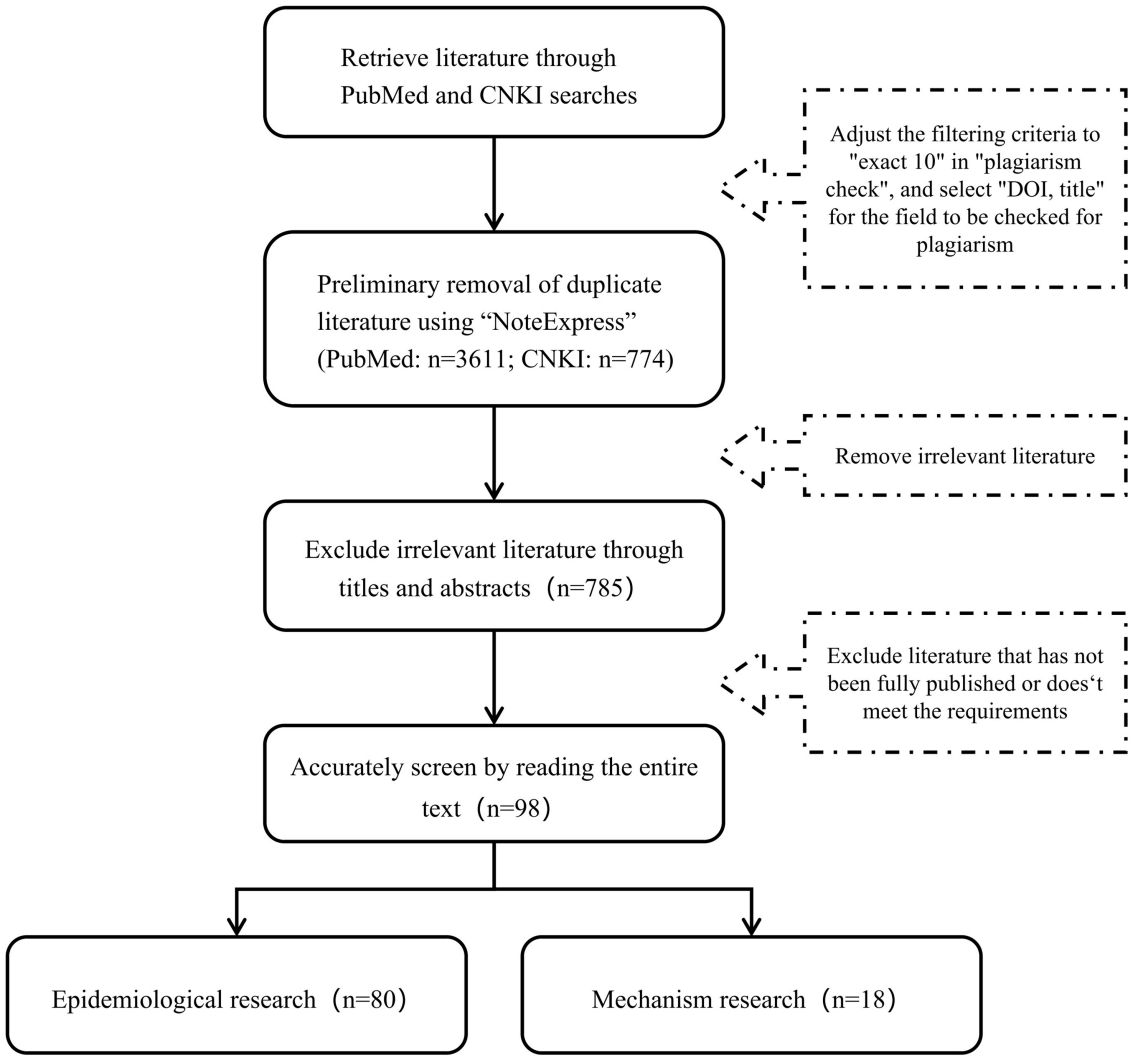
Literature screening process diagram.

After eliminating duplicates using the document management software “NoteExpress,” a total of 4,385 documents were retrieved. Further screening was performed based on titles, abstracts, and full texts according to following inclusion and exclusion criteria. Inclusion criteria: the literature reported the results of independent epidemiologic studies or experimental results; the study exposure factor was PPCPs pollutants; for epidemiological studies, the study outcome was AD, and for mechanism studies, clear AD evaluation specific indicators (such as Th2 related inflammatory factors, etc.) should be used; there were clear indicators describing the risk, such as odds ratio (OR), hazard ratio (HR), relative risk (RR), and so on. Exclusion criteria: those who did not meet the inclusion criteria; the research types were non original studies such as meta-analysis, systematic review, commentary, letters, conference abstracts, etc.; there were obvious deficiencies in the research data, such as missing key information, incorrect experimental methods, or contradictory conclusions; AD was only mentioned incidentally in the multiple outcome analysis, and no targeted analysis was conducted on the association between PPCPs and AD. In order to provide a more comprehensive overview of the status in this field, we did not remove small sample studies. We emphasized the limitations caused by this choice in “Factors interfering with the results.” Finally, 80 epidemiologic studies and 18 mechanism experiments were included for further consideration (Figure 1). The screening process was conducted independently by two researchers, with the final results synthesized from both.

## 3 Results of epidemiological research

**Figure 2:**
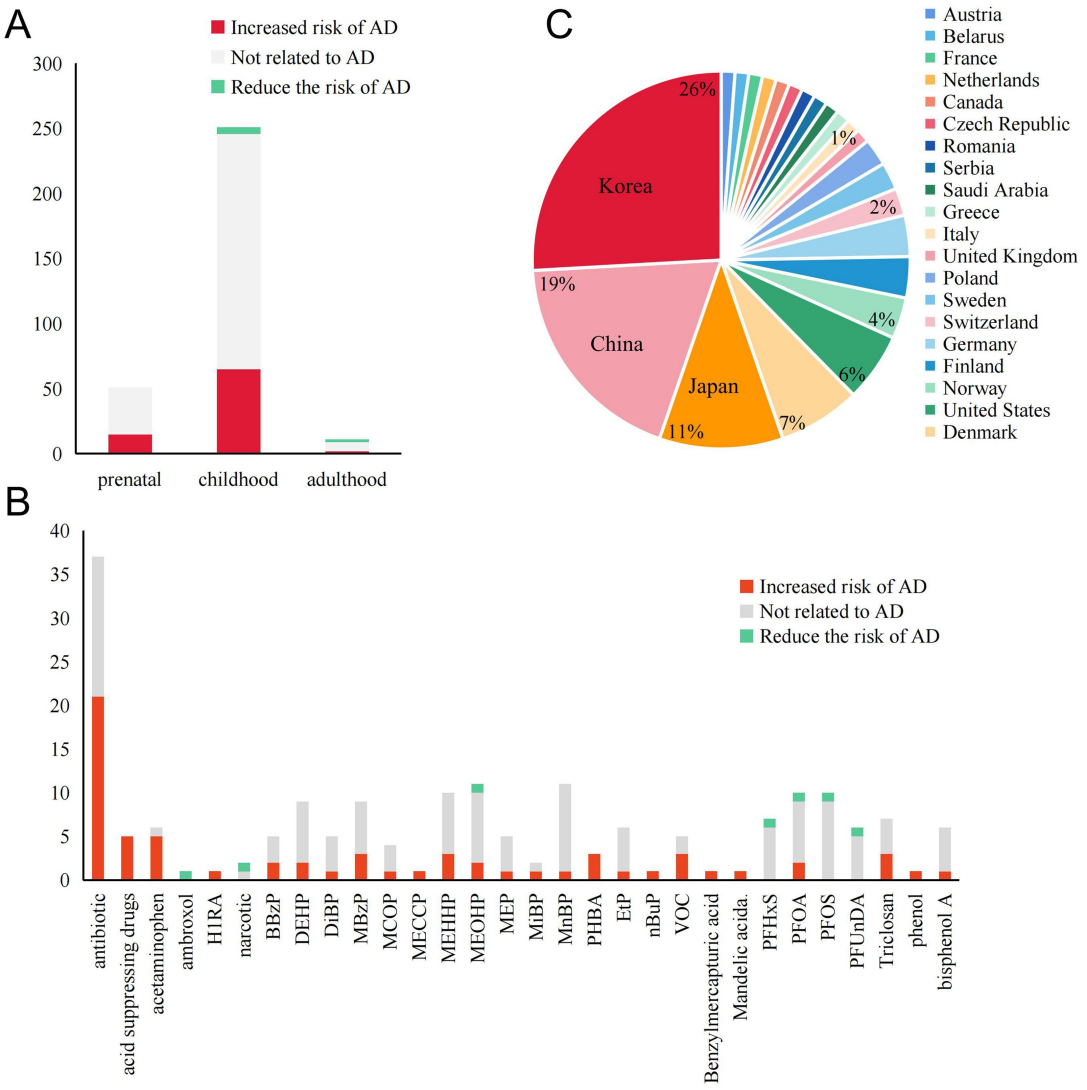
Summary of exposure outcomes, exposure windows, and geographical areas involved in epidemiological studies. **(A)** The relationship between exposure windows and AD outcomes at different stages of life. **(B)** The number of studies supporting or opposing the role of various PPCPs in AD. PPCPs without any reported positive results are not included. **(C)** The regions involved in epidemiological research and the proportion of those regions mentioned.

Our research includes studies from Asia (mainly from Southeast Asia like China, Japan, and Korea), Europe (involving 17 countries), and North America (the United States, Canada) (Figure 2C). Another study involving 22 countries worldwide will not be elaborated on here. We compiled the results of the correlation between PPCPs exposure and AD at different stages of life. Figure 2A shows that most studies discuss the dangers of exposure during pregnancy and adolescence. Nearly a quarter of the results are positive, indicating that the pathogenic risk of PPCPs cannot be disregarded. Very few research has been conducted on adults. Antibiotics (ABX) and phthalates are the most frequently reported PPCPs, according to further pollution classification (Figure 2B). These pollutants affect human health by increasing the risk of AD or worsening the condition of AD. In addition, mechanism studies have explored the biological mechanisms of the harm caused by the above-mentioned pollutants, further supporting their risk in AD pathogenesis. Therefore, we consider ABX and phthalates as common high-risk PPCPs in AD patients. Fluorides are the primary subject of reports on the protective effects of contaminants. Although not all studies yield positive evidence, there is broad agreement among scholars that PPCPs are risk factors. Conflicting results may arise from differences in exposure concentrations, times, and populations.

### 3.1 Pharmaceutical

#### 3.1.1 Antibiotics

AD patients are prone to infection, making ABX a critical therapeutic intervention (15). However, there is growing evidence that early-life ABX exposure increases the risk of AD. Only two of the listed studies covered youths aged 18–20. The remaining research subjects are all pediatrics (Table 1). It should be alert to the occurrence of AD due to maternal and early life antibiotic therapy.

**Table 1:** The correlation between ABX exposure and the risk of AD.

Pollutant	Method	Country/Region	Population	Exposure period	Sample size	Correlation	Risk indicator; 95%CI	P-value	Ref.
ABX	Cohort study	Olmsted County	0–14 Y	0–2 Y	14,166	+	H 1.47; 1.12–1.94	0.006	(24)
ABX	Prospective cohort study	Sweden	0–9 Y	Intrauterine	722,767	+	H 1.10; 1.09–1.12	/	(28)
				0–1 Y		+	H 1.52; 1.50–1.55	/	
ABX	Prospective cohort study	Copenhagen	0–13 Y	Intrauterine	411	−	O 1.0; 0.4–2.2	0.96	(136)
ABX	Multi-central cross-sectional study	22 countries	6–7 Y	0–1 Y	120,799	+	O 1.41; 1.34–1.48	/	(137)
ABX	Cross-sectional study	Korea	0–19 Y	7 years before onset	5,626,328	+	O 10.446; 10.111–10.792	/	(138)
ABX	Case–control study	Hong Kong	4 M-3 Y	Intrauterine or breastfeeding period	206	+	O 2.276; 1.151–4.504	/	(30)
ABX	Retrospective cohort study	Elizabeth Town	2 Y	At birth	492	−	R 1.03; 0.75–1.41	0.854	(16)
				0–2 Y		−	R 1.26; 0.90–1.77	0.163	
ABX	Prospective cohort study	Shanghai	5 Y	Intrauterine	251	−	O 1.10; 0.55–2.17	/	(139)
ABX	Prospective cohort study	Tokyo	5 Y	0–2 Y	1,196	+	O 1.40; 1.01–1.94	0.044	(38)
ABX	Cohort study	Southampton	6 M	Early intrauterine	3,158	−	O 0.87; 0.70–1.08	0.21	(35)
			12 M			−	O 0.97; 0.80–1.18	0.79	
			6 M	Late intrauterine		−	O 1.06; 0.89–1.25	0.51	
			12 M			−	O 1.10; 0.93–1.30	0.27	
			12 M	0–1 Y		+	O 1.59; 1.23–2.07	0.001	
ABX	Retrospective cohort study	Bethesda	4.6 Y (Me)	0–6 M	792,130	+	H 1.18; 1.16–1.19	/	(41)
ABX	Retrospective cohort study	Taiwan	0–10 Y	Intrauterine	1,288,343	+	H 1.04; 1.03–1.05	<0.001	(21)
ABX	Cohort study	Italy	1–14 Y	0–1 Y	73,816	−	H 1.02; 0.97–1.07	/	(140)
ABX	Nested case–control study	Taiwan	1.3 Y (Me)	Intrauterine	21,816	−	O 1.05; 0.98–1.12	0.147	(25)
				Lifetime		+	O 1.67; 1.55–1.79	<0.001	
ABX	Prospective cohort study	Korea	3 Y	0–6 M	1,637	+	O 1.40; 1.09–1.81	0.001	(31)
ABX	Case–control study	Korea	0–4 Y	Lifetime	2,283,601	+	O 1.11; 1.09–1.13	/	(32)
ABX	Cross-sectional study	Southern Romania	0–18 Y	Intrauterine	1,046	−	O 1.28; 0.99–1.65	0.06	(17)
				0–1 Y		−	O 1.33; 0.90–1.97	0.15	
ABX	Cohort study	Japan	1 Y	Intrauterine	70,408	−	O 1.01; 0.97–1.06	0.589	(141)
ABX	Prospective cohort study	Countryside of Austria, Finland, France, Germany, Switzerland	0–6 Y	Intrauterine	1,080	−	O 1.19; 0.69–2.05	/	(33)
				0–1 Y	1,019	+	O 2.65; 1.69–4.16	/	
ABX	Retrospective cohort study	Changsha, Wuhan, Xiamen, Urumqi, Hohhot	Freshman	0–7 Y (i.v.)	20,123	+	R 1.36; 1.14–1.62	/	(142)
				0–7 Y (PO)		+	R 1.18; 1.01–1.38	/	
Penicillin	Cohort study	Britain	0–17 Y	Intrauterine	1,023,140	+	H 1.43; 1.41–1.44	/	(22)
				0–90 D		+	H 1.70; 1.67–1.73	/	
Cephalosporin				Intrauterine		+	H 1.35; 1.32–1.37	/	
				0–90 D		+	H 1.70; 1.56–1.85	/	
Sulfonamide				Intrauterine		+	H 1.24; 1.20–1.29	/	
				0–90 D		+	H 1.46; 1.37–1.56	/	
Macrolides				Intrauterine		+	H 1.36; 1.32–1.40	/	
				0–90 D		+	H 1.77; 1.69–1.86	/	
ABX	Cohort study	Denmark	18 M	Intrauterine	41,895	−	O 1.01; 0.83–1.22	/	(23)
ABX	Prospective cohort study	Greek	18 M	Intrauterine period (i.v.)	236	+	O 7.70; 1.23–48.27	0.03	(143)
ABX	Cohort study	Japan	0–3 Y	Intrauterine	78,678	−	O 1.02; 0.97–1.08	/	(144)
ABX	Cohort study	Korea	0–2 Y	/	202,616	+	O 1.07; 1.06–1.09	/	(34)
			≥2 Y	/	55,003	+	O 8.78; 7.42–10.04	/	
ABX	Cross-sectional study	Taipei	6–8 Y	0–1 Y	24,999	+	O 1.37; 1.22–1.53	<0.001	(37)
ABX	Cross-sectional study	Korea	6–7 Y	Babyhood	4,003	−	O 1.09; 0.88–1.39	/	(145)
			12–13 Y		4,112	−	O 1.04; 0.86–1.34	/	
ABX	Case–control study	Finland	2 Y	At birth	433	+	O 2.21; 1.20–4.10	0.010	(146)
				0–6 M		+	O 0.50; 0.26–0.95	0.03	
ABX	Retrospective cohort study	Japan	1–10 Y	0–1 Y	85,954	+	H 1.12; 1.04–1.21	/	(39)
ABX	Cross-sectional study	Shanghai	4–6 Y	0–1 Y	13,335	+	O 1.17; 1.05–1.31	0.005	(147)
ABX	Cohort study	Taiwan	0–12 Y	Intrauterine	900,584	+	H 1.12; 1.11–1.13	/	(29)
ABX	Prospective cohort study	Canada	5 Y	0–1 Y	2,484	+	O 1.81;1.28–2.57	<0.001	(26)
ABX	Cohort study	Korea	2–14 Y	0–24 M	4,069,771	+	H 1.33; 1.27–1.39	/	(27)
ABX	Prospective cohort study	Southwest Finland	8.1 Y (Me)	At birth (no infection)	11,255	−	O 1.25; 0.94–1.68	/	(148)
				At birth (with infection)		+	1.49; 1.15–1.94	/	
				0–6 M		+	1.38; 1.15–1.64	/	
ABX	Cross-sectional study	Saudi Arabia	6–7 Y	0–1 Y	3,614	−	O 1.42; 0.93–2.17	0.109	(44)
quinolones	Retrospective cohort study	Korea	0–9 Y	Early intrauterine	168,730	−	R 1.04; 1.02–1.05	/	(149)

Y: years old; M: months old; D: days old; H: Hazard Ratio; O: Odds Ratio; R: Relative Risk;+: Increased risk of AD; −: not related to increased risk of AD onset; Me: median; i.v.: intravenous injection; PO: take orally. The same applies to the subsequent tables below.

Our review identified thirteen epidemiological studies only involved intrauterine exposure. The results on maternal exposure are not uniform. Four studies suggested that intrauterine exposure to ABX increased the risk of AD in offspring. The remaining studies, however, failed to identify positive results. Interestingly, two of these studies revealed potential hazards through further analysis. Subgroup analyses indicated that exposure lasting more than 24 h during delivery (RR 1.99, 95%CI 1.13–3.49, *p* = 0.017), intravenous administration, and exposure in late pregnancy (OR 2.94, 95%CI 1.21–7.12, *p* = 0.02) posed key risks (16, 17). This is consistent with the sensitivity during the critical period of late fetal skin barrier development (about 28 weeks of pregnancy). At this point, the fetal skin begins to keratinize, and ABX entering through the placenta are more likely to interfere with the expression of barrier related genes (18–20). In addition, the role of genetic background is not yet clear, with some studies implying that ABX induction is more pronounced in participants without an atopic genetic background (the mothers of the participants suffer from asthma, hay fever, food allergies, or eczema) (21, 22), but conclusions are not uniform (23).

Exposure during infancy and early childhood is of equal concern. Almost all studies focus on the exposure risk before the age of seven (21/22), with 16 papers explicitly stating that their study evaluated exposure before the age of two, implying that the age range of 0–2 is a particularly sensitive window for ABX exposure. In addition, studies demonstrated that the earlier the exposure to ABX, the stronger the positive correlation with AD. A U. S. cohort study (24) showed that antibiotic exposure during 0–6 months carried a higher risk than 6–12 and 12–24 months. A case–control study from Taiwan (25) also found that the risk of antibiotic exposure was greater at 0–1 year (OR 1.40, 95%CI 1.31–1.49, *p* < 0.001) or 1–2 years of age (OR 1.33, 95%CI 1.22–1.43, *p* < 0.001) compared to exposure after 2 years of age. Similar conclusions have also been proven by Courtney (26) and Seong (27). Statistics have found that compared to intrauterine exposure, early life ABX exposure has a higher pathogenicity. Only four studies stated that they failed to find any risks.

Increased exposure frequency of ABX in utero and infancy (higher risk of multiple exposures) (26–34) and narrow-spectrum ABX (28) posed a greater risk of AD. Even more interesting is that an “inverse U-shaped” dose dependence was observed in two studies, indicating that the pathogenic risk of antibiotics decreases after reaching a certain critical value (24, 35). This may be related to the adaptability of the body’s immune regulation (36). Sex differences also affect outcomes, with Aversa’s (24) study indicating that early-life antibiotic use increases AD risk predominantly in girls (HR 1.50, 95% CI 1.00–2.24), while another study (37) suggesting that boys aged 6–8 have a higher risk due to first-year antibiotic exposure (OR 1.11, 95% CI 1.02–1.20, *p* = 0.012). Macrolides, commonly used in AD patients, are considered as high-risk ABX (22, 38, 39). Interestingly, macrolide ABX reduce the risk of AD in girls, while cephalosporins exert a positive effect (24).

Overall, the AD-inducing effects of ABX are widely recognized. However, most studies did not test the residual antibiotic levels *in vivo*. Pharmacokinetic differences due to individual differences affect the half-life of drugs, which makes further concentration detection necessary (40). Besides, questionnaires or statistical generalization of prescribed medications are the main ways of assessing exposure. But it does not reflect the route of exposure. Environmental trace exposures are also overlooked. Notably, ABX use after infection reduces the increased risk of AD caused by the infection itself (32, 41).

#### 3.1.2 Other drugs

Among the retrieved literature, except for one article mentioning the impact of ASM on AD pathogenesis in individuals aged 18 and above (which yielded positive results), other studies still with an eye on the pathogenicity of the drug in children. Overall, the number of studies investigating the effects of other drugs on AD is limited (Supplementary Table S1). Among them, acid-suppressive medication (ASM) and acetaminophen (AP) are considered drugs with high risk of AD.

Among the five retrieved literature, four suggested that children exposed to ASM have a higher susceptibility to AD, and one study found the AD pathogenicity of ASM in adult. Exposure to ASM during late pregnancy was associated with AD (HR 1.68, 95%CI 1.17–2.41), while exposure during early pregnancy was not (42). As common ASM, histamine 2 receptor antagonists (H2RAs) contributed a major role in the pathogenesis of AD, rather than proton pump inhibitors (PPIs). Five of six studies showed an increased risk of AD caused by AP. Li et al. (43) stated that exposure to AP in early (OR 1.16, 95%CI 1.05–1.28) and mid pregnancy (OR 1.14, 95%CI 1.03–1.27) increased the likelihood of AD in offspring with a dose accumulation effect. According to studies from Saudi Arabia (44) and Kosovo (45), exposure to AP at least once a month within the year preceding onset can lead to the onset of AD.

Surprisingly, the use of anesthetics reduces the risk of AD. In Kuo’s study (46), anesthetic exposure was a protective factor for childhood AD (HR 0.60, 95%CI 0.53–0.69). This protective effect is not affected by the anesthesia method. Kim’s study (47) distinguished the types of anesthetics and found that the risk of AD was only reduced when using thiopental injection. However, as we only found two relevant studies, the reliability of the conclusions still needs to be verified.

Tacrolimus, histamine H1 receptor antagonist and the phlegm chemotherapeutic agent ambroxol were also found to be pathogenic. But only one study provided relevant results, which makes the conclusion contingent. Most clinically used drugs were not considered. Although some drugs were not found to be associated with AD for the time being, we cannot ignore the positive associations mentioned in some studies. More studies are needed to prove the safety of the drugs.

### 3.2 Personal care products

#### 3.2.1 Phthalates

Phthalates, universally used in detergents, lubricants, and beauty products, can harm the body by penetrating the respiratory system and skin (48). Previous studies have focused on the correlation between phthalates and allergic diseases. Fourteen related studies were taken into consideration (Table 2; to ensure coherence in the writing, we have placed the correspondence between abbreviations and complex full names at the end of the table). Although the current research is limited in quantity, the results are still dazzling based on the rich variety and metabolites of phthalates.

**Table 2:** The correlation between phthalates exposure and the risk of AD.

Pollutant	Concentration ^a.^	Method	Country/Region	Population	Sample	Sample size	Correlation	Risk indicator; 95%CI	P-value	Ref.
MEHHP	13.12; 14.57 (Q3) ^b.^	Cross-sectional study	Korea	12–17 Y	Urine	797	−	O 1.38; 0.86–2.22	/	(56)
MEOHP	9.28; 10.82						−	1.37; 0.85–2.22	/	
MECCP	23.24; 26.76						+	1.81; 1.16–2.80	/	
MnBP	34.29; 41.69						−	1.17; 0.66–2.08	/	
MBzP	3.18; 4.24						+	1.81; 1.01–3.25	/	
MCOP	1.53; 1.64						−	1.32; 0.77–2.26	/	
MCNP	0.41; 0.43						−	1.05; 0.66–1.66	/	
MCPP	1.21; 1.32						−	1.15; 0.71–1.86	/	
MnBP	0.04142 (GM)	Cross-sectional study	Korea	3–17 Y	Urine	2,208	−	O 1.08; 0.89–1.31	/	(54)
MBzP	0.00282						+	1.15; 1.01–1.30	/	
MCOP	0.00187						+	1.35; 1.02–1.78	/	
MCNP	0.00049						−	1.20; 0.95–1.52	/	
MCPP	0.00158						−	1.01; 0.79–1.31	/	
DEHP	0.07869						+	1.39; 1.09–1.79	/	
MEHHP	0.02262						+	1.26; 1.01–1.59	/	
MEOHP	0.01560						+	1.38; 1.14–1.67	/	
MECPP	0.03763						−	1.26; 0.96–1.65	/	
MEOHP	0.00803 (Me)	Prospective cohort study	Poland	9 Y	Urine	145	Re	O 0.49; 0.27–0.87	0.02	(55)
MBzP	0.00195						−	1.40; 0.89–2.20	0.15	
MnBP	0.10888						−	0.91; 0.56–1.48	0.70	
MEHHP	0.02034						+	1.90; 1.18–3.05	0.008	
MEHP	0.00304						−	0.85; 0.57–1.25	0.410	
MEP	0.10507						−	1.17; 0.74–1.86	0.497	
MEP	50.5; 60 (IQR; ng/mg)^b.^	Prospective cohort study	Leipzig	0–3 Y	Urine (intrauterine)	610	−	O 1.45; 0.75–2.83	0.268	(52)
MiBP	62.4; 73.3						+	2.21; 1.10–4.45	0.026	
MnBP	96.9; 112.7						−	1.79; 0.91–3.52	0.090	
MBzP	6.4; 6.9						−	1.28; 0.65–2.52	0.470	
MEHP	7.2; 7.7						−	1.50; 0.76–2.98	0.238	
MEP	0.0158; 0.0198 (GM)^b.^	Cross-sectional study	Denmark	3–5 Y	Urine	440	+	O 2.27; 1.12–4.62	/	(59)
MnBP	0.0822; 0.0800						−	0.62; 0.60–2.39	/	
MiBP	0.0702; 0.0674						−	0.97; 0.48–1.94	/	
MBzP	0.0137; 0.0131						−	1.43; 0.72–2.88	/	
MEHP	0.0048; 0.0043						−	0.93; 0.46–1.87	/	
MEHHP	0.0341; 0.0300						−	0.68; 0.34–1.36	/	
MEOHP	0.0171; 0.0149						−	0.79; 0.39–1.60	/	
MECPP	0.0388; 0.0347						−	0.78; 0.39–1.56	/	
MEOHP	71.97 (GM; μg/g Cr)	Longitudinal study	Seoul	3–7 Y (male)	Urine	18 (460 times)	−	O 2.28; 0.74–7.00	/	(61)
MEHHP	68.11						−	1.21; 0.47–3.07	/	
MnBP	76.02						*	2.85; 1.12–7.26	/	
MEP	61.7582 (μg/g Cr)	Prospective cohort study	Taiwan	2 Y	Urine (at the age of 2)	218	−	O 1.33; 0.52–3.41	/	(58)
				5 Y			−	1.82; 0.68–4.86	/	
MBP	237.9412			2 Y			−	0.75; 0.29–1.93	/	
				5 Y			−	0.80; 0.31–2.05	/	
MBzP	8.2000			2 Y			+	2.50; 1.08–5.79	/	
				5 Y			−	1.98; 0.81–4.87	/	
MEHP	71.3208			2 Y			−	1.31; 0.50–3.45	/	
				5 Y			−	1.76; 0.67–4.64	/	
DMP	<MDL^c.^	Cross-sectional study	Japan	Locals	dust	516	−	O 2.56; 1.00–6.55	0.991	(49)
	<MDL						−	0.36; 0.08–1.52	0.069	
DEP	0.28 (Med; μg/g dust)						−	1.14; 0.46–2.77	0.401	
	0.26						−	1.24; 0.50–3.11	0.705	
DiBP	2.4						+	4.84; 1.46–16.00	0.010	
	1.9						−	1.29; 0.57–2.95	0.541	
DnBP	19.3						−	1.19; 0.46–3.07	0.714	
	20.6						−	1.02; 0.38–2.76	0.966	
BBzP	1.9						+	5.46; 2.06–14.48	0.001	
	1.7						−	1.06; 0.43–2.62	0.902	
DEHP	759						+	2.60; 1.07–6.30	0.035	
	854						−	1.93; 0.74–5.02	0.175	
DiNP	95						−	1.22; 0.54–2.75	0.633	
	92.3						−	0.88; 0.41–1.90	0.740	
DEHA	4.6						−	2.32; 0.90–6.03	0.900	
	5.4						−	0.92; 0.35–2.37	0.984	
DnBP	0.98 (Me; mg/kgBW/day)^e.^	Case–control study	Funan Island	3–5 Y	Urine	500	−	/	/	(57)
DiBP	1.89						−	/	/	
BBzP	0.029						+	O 2.8; 1.17–6.7	/	
DEHP	0.83						−	/	/	
MEHHP	0.01051 (GM)	Multi-central prospective cohort study	Korea	6 M	Urine (early intrauterine)	413	−	Rd 0.042; (−0.160)-0.244	/	(51)
MEOHP	0.01015						−	(−0.036); (−0.251)-0.180	/	
MnBP	0.03239						−	0.006; (−0.139)-0.151	/	
MEHHP	0.0130				Urine (late intrauterine)		+	0.290; (−0.043)-0.623	/	
MEOHP	0.0116						+	0.035; (−0.288)-0.116	/	
MnBP	0.03339						−	(−0.059); (−0.234)-0.116	/	

Rd: Risk difference; Re: Reduce the risk of AD; *: associated with worsening symptoms of AD; GM: geometric mean; DEHP: Di(2-ethylhexyl) phthalate; DINP: di-isononyl phthalate; DMP: di-methyl phthalate; DnBP: di-n-butyl phthalate; MEHHP: mono(2-ethyl-5-hydroxyhexyl)phthalate; 7OH-MiNP: mono(4-methyl-7-hydroxyoctyl)phthalate; 7oxo-MiNP: mono(4-methyl-7-oxo-octyl)phthalate; BBzP: butyl benzyl phthalate; DEP: di-ethyl phthalate; DiBP: di-isobutyl phthalate; DOP: di-n-octyl phthalate; MBP: mono-n-butyl phthalate; MBzP: monobenzyl phthalate; MCNP: monocarboxyononyl phthalate; MCOP: monocarboxyoctyl phthalate; MCPP: mono(3-carboxypropyl)phthalate; MECCP: mono(2-ethyl-5-carboxypentyl)phthalate; MEHP: mono-(2-ethylhexyl)phthalate; MEOHP: mono(2-ethyl-5-oxohexyl)phthalate; MEP: monoethyl phthalate; MiBP: monoisobutyl phthalate; MnBP: mono-n-butyl phthalate. a: The concentration unit of unmarked pollutants is μg/mL. b.: The first number is for non AD patients, and the last number is for AD patients. c.: The number above represents the pollutant concentration of ground dust, while the number below represents the pollutant concentration of indoor dust. e.: Total intakes. These literature (54, 69, 134, 150) did not find any positive results (Supplementary Table S2).

Thirteen studies focused on the population under 18 years old. And one study did not emphasize the age of the participants. In this study, higher concentrations of DiBP (OR 4.84, 95%CI 1.46–16.00), BBzP (OR 5.46, 95%CI 2.06–14.48) and DEHP (OR 2.60, 95%CI 1.07–6.30) in ground dust were found to be associated with AD incidence in the local long-term population. After age stratification, participants under the age of 14 have higher sensitivity to pollutant exposure because they can be infected by lower concentrations (49). It may be attributed to youngsters’ higher exposure to floor dust and more time spent at home, where phthalate concentrations in indoor air are higher (48, 50). Two studies involve cross-generational exposure. The results showed that higher concentrations of MiBP, MEHHP and MEOHP in maternal urine were associated with the onset of AD in offspring (51, 52). Nine out of the remaining ten studies had participants over the age of 2. The positive correlation between DEHP (metabolites MEHHP, MECCP, MEOHP) (51, 53–56) and BBzP (metabolite MBzP) (49, 54, 57, 58) and AD is most prominent. However, a longitudinal study in Poland (55) found that higher current urinary concentrations of MEOHP reduced the risk of AD in 9-year-old children (OR 0.49, 95%CI 0.27–0.87). MCOP (54), MEP (59), and MiBP (52) have also been found to be potentially correlated with the development of AD.

Although most of these pollutants have only been found to be associated with AD pathogenesis in a single study (except for MEHHP and MBzP), DEHP and BBzP, as the main types of pathogenic pollutants discovered, still need to be taken seriously. Inconsistent findings may be caused by phthalates’ short half-life in the body (60). A Taiwanese prospective cohort study (58) showed that a high concentration of MBzP (8.20 μg/g Cr) in urine at 2 years of age is positively associated with the incidence of AD at the same age (OR 2.50, 95%CI 1.08–5.79), but does not increase the risk of AD at 5 years of age. In a study by Kim et al. (61), aggravation of AD symptoms was related to an increase in MnBP levels in the urine on the same day (OR 2.85, 95%CI 1.12–7.26) and in the previous day (OR 2.74, 95%CI 1.21–6.20). MEOHP levels in urine 2 days before were positively linked with AD symptoms (OR 3.11, 95%CI 1.01–9.61). These results indicate that a single urinary test is not a reliable sign of exposure level. The environmental endocrine disruptor effect of phthalates is also a significant factor. Lee S et al. (51) showed that when considering the impact of gender, MEOHP was only pathogenic to girls (OR 1.84, 95%CI 1.20–2.88). And MEHHP was more harmful to girls than boys (girls: OR 1.96, 95%CI 1.30–3.06; boys: OR 1.61, 95%CI 1.02–2.62). In summary, out of the fourteen studies included, ten proposed the discovery that certain phthalates or their metabolites have AD pathogenicity. However, for a single metabolite, negative results still dominate. Existing research on pollutant concentration based on a single time point cannot effectively reflect the pathogenic effects of phthalates. More longitudinal research is needed to uncover possible mechanisms.

#### 3.2.2 P-hydroxybenzoic acid

P-hydroxybenzoic acid (PHBA), as a bacteriostatic agent, is widely used in cosmetics and pharmaceuticals (62–64). PHBA’s immunomodulatory function may raise the risk of allergy disorders (65). Another proposed harmful mechanism is the induction of oxidative stress (66). Five studies, all with subjects under the age of 15, explored the association between PHBA and AD and found evidence of PHBA pathogenicity (Table 3).

**Table 3:** The correlation between PHBA exposure and the risk of AD.

Pollutant	Concentration ^a.^	Method	Country/Region	Population	Sample	Sample size	Correlation	Risk indicator; 95%CI	P-value	Ref.
PHBA	83.6; 192 (Me; pmol/mg Cr)^b.^	Cross-sectional study	Shiga Town	0–3 Y	Urine	236	+	Exp (β)4.995; 2.248–11.099	<0.001	(151)
MeP	0.0382 (IQR)	Prospective cohort study	Leipzig	0–8 Y	Urine (intrauterine)	261	−	O 1.28; 0.90–1.83	0.174	(68)
EtP	0.00250						+	1.44; 1.04–2.00	0.029	
nPrP	0.00460						−	1.04; 0.69–1.56	0.856	
iBuP	0.00017						−	1.39; 0.99–1.93	0.054	
nBuP	0.00070						+	1.95; 1.22–3.12	0.005	
propyl hydroxybenzoate	/	Cross-sectional study	Korea	10–12 Y	Urine	455	−	O 1.479; 0.673–3.249	0.330	(152)
MeP	0.0463 (GM)	Cross-sectional study	Korea	3–5 Y	Urine	571	−	odds 1.10; 0.93–1.29	/	(69)
EtP	0.0142						−	1.18; 0.93–1.50	/	
PrP	0.00436						−	1.11; 0.997–1.24	/	
MeP	0.0289			6–11 Y			−	1.01; 0.86–1.18	/	
EtP	0.0114						−	1.02; 0.89–1.17	/	
PrP	0.00183						−	1.03; 0.88–1.20	/	
PHBA	8.3 (Me; pmol/Cr)	Cross-sectional study	Tokyo, Japan	0–15 Y	Urine	138	+	O 4.610; 1.230–17.300	/	(67)
MeP	15.35	Cross-sectional study	Korea	3–5 Y	Urine	556	−	O 0.87; 0.72–1.04	/	(153)
	13.89 (GM; μg/g Cr)			6–11 Y		701	−	1.05; 0.90–1.21	/	
	7.47			12–17 Y		731	−	1.11; 0.92–1.33	/	
EtP	32.6			3–5 Y		556	−	0.99; 0.77–1.28	/	
	15.08			6–11 Y		701	−	1.00; 0.89–1.13	/	
	31.41			12–17 Y		731	−	0.96; 0.79–1.17	/	
PrP	0.8			3–5 Y		556	−	0.96; 0.84–1.10	/	
	0.97			6–11 Y		701	−	1.07; 0.97–1.18	/	
	0.35			12–17 Y		731	−	1.14; 0.98–1.32	/	
BuP	0.73			3–5 Y		556	−	0.44; 0.12–1.65	/	
	0.49			6–11 Y		701	−	1.06; 0.70–1.59	/	
	0.45			12–17 Y		731	−	0.82; 0.50–1.36	/	

IQR: interquartile range; MeP: methylparaben; EtP: ethylparaben; nPrP: n-propylparaben; iBuP: isobutylparaben; nBuP: n-butylparaben; BuP: butylparaben; PrP: propylparaben. a.: The concentration unit of unmarked pollutants is μg/mL. b.: The first number is for non AD patients, and the last number is for AD patients.

There are three studies exploring the effect of total PHBA on AD, all of which have found positive evidence. A cross-sectional studies from Korea (67) did not find direct evidence, but they claimed that PHBA was associated with the exacerbation of skin AD symptoms such as redness, swelling, and peeling. Thürmann et al. (68) examined different types of PHBA and found that only intrauterine exposure to EtP and nBup increased the risk of AD in children aged 0–8 years. Interestingly, maternal history of AD protected the kids from intrauterine exposure to EtP or nBuP. Gender and age also matter. Younger boys seem to be more susceptible to PHBA. PrP increased the risk of AD in boys aged 3–5 years (odds1.23, 95%CI 1.08–1.41) and girls aged 6–11 years (odds 1.22, 95%CI 1.001–1.48), whereas MeP (odds 1.26, 95%CI 1.06–1.49) and combined exposure to MeP + PrP (odds1.85, 95%CI 1.28–2.70) were only associated with AD in boys aged 3–5 years (69).

Most studies show the effect of total PHBA exposure, but only a handful may contribute. The type of PHBA used in various regions may produce inconsistent results. Further categorizing of PHBA is necessary to determine the true causal cause. Besides, studies on total PHBA exposure have smaller sample sizes, resulting in less reliable conclusions. Large-scale longitudinal cohort studies are required based on the risks associated with pollutant exposures.

#### 3.2.3 Fluoride

Fluoride, present in dental care products, has a controversial association with allergic diseases, including AD (70, 71). Nine studies were included in the analysis. Among them, three studies found that fluoride may be a protective factor for AD, while two studies believed that fluoride exposure increased the incidence rate of AD. The remaining four studies found no significant results (Supplementary Table S3).

PFOA and PFOS are the main compounds discussed (72). Three studies in children showed that intrauterine exposure to PFOA increases the prevalence of AD (73–75). However, in a prospective cohort study of adults (76), PFOA (OR 0.58, 95%CI 0.37–0.90) and PFOS (OR 0.56, 95%CI 0.32–0.95) was negatively associated with AD. PFHxS (RR 0.79, 95%CI 0.34–0.99) (72) and PFUnDA (OR 0.69, 95%CI 0.55–0.86, *p* = 0.001) (77) also were found to be protective factors. We noticed that the age of the subjects seemed to be higher in studies that found protective effects than in studies that indicated harmful effects. This suggests that young participants seem to be more susceptible to fluoride damage. But these injuries will weaken with age. The dual effects of immunosuppression and chronic inflammation induced by fluoride may be the key to this result (78, 79). Besides, female seem to be more susceptible to fluoride, as several pollutant-AD associations are only observed in girls (72, 74, 76, 77). It may be caused by differences in sex hormones and menstrual bleeding (72, 76). Some other compounds are mentioned, with varying associations. This could be owing to the fact that fluorides are a complicated family, with fluorides with longer carbon chains and more side chains exhibiting lower removal effectiveness (72, 77).

#### 3.2.4 Other PCPs

Bisphenol A (BPA) is an important industrial raw material widely used in plastic medicine bottles, cosmetic packaging, and the production of fine chemical products such as ultraviolet absorbers and fungicides (80). Four studies mentioned the role of BPA and its analogues in pathogenesis of AD. Only one cross-sectional study from Korea (51) proved that BPA affected on children aged 6–11 years (odds 1.31, 95%CI 1.06–1.61). Subanalysis by gender revealed that BPA caused AD only in boys (odds 1.34, 95%CI 1.05–1.69). A one-year longitudinal study (61) showed that AD symptoms in boys aged 3–7 years did not respond to same-day urinary BPAG levels but worsened with previous day’s levels (OR 2.01, 95%CI 1.08–3.74). These pieces of evidence suggest that bisphenol substances cause greater damage to boys. The possible mechanism is currently unclear. Considering the potential link between BPA exposure and the risk of AD in boys, a more in-depth BPA risk assessment is necessary for preventing AD.

Volatile organic compounds (VOC), found in perfume, nail polish, hair gel and household cleaners (81), which has been found to promote the onset of AD in all five studies. VOC has a promoting effect on the onset and deterioration of AD in both adults and children. However, all five studies included were from Korea, which undoubtedly increases the limitations of the results. As a general term for a type of pollutant, VOC has a complex and diverse composition. The exposure level of total VOC cannot reflect the actual pollutants at play. Further research indicates that Benzylmercapturic acid and Mandelic acid were a possible pathogenic agent, while no adverse effects have been found for other components (82).

Triclosan (TCS) is also a compound of interest because of its widespread use as a deoderant. We retrieved three relevant literatures, two of which suggest that TCS exposure is a risk factor for AD. In the study that did not yield a positive result, the content of TCS in urine has not reached the detection limit (67). Extremely low levels of pollutants may be the cause of negative results. Therefore, we still need to pay attention to the potential inflammatory risks of TCS.

Phenol is an important raw material for the production of certain fungicides and preservatives. AD can be categorized into the IgE-high, extrinsic subtype and the IgE-normal, intrinsic subtype. Some of the studies mentioned earlier have revealed that some pollutants are interrelated to AD with abnormal total IgE (54). However, they did not make a clear distinction about the association among pollutants, IgE and AD. A cohort study from Minsk demonstrated that phenol has different pathogenic effects on AD with different phenotypes. Higher mean yearly phenol exposure concentrations were linked to AD (OR 1.724, 95%CI 1.091–2.723, *p* = 0.020). As for intrinsic AD, phenol is considered a protective factor(OR 0.029, 95%CI 0.004–0.194, *p* < 0.001) among infants aged 0–2 years (83). It confirms that subtyping analysis of AD may be the key to explaining some conflicting results.

In conclusion, the association between PPCPs and AD is complex (Supplementary Table S4), with various factors influencing the risk. Further research is needed to clarify these relationships and inform public health interventions.

## 4 Possible pathogenic mechanisms of PPCPs

**Figure 3:**
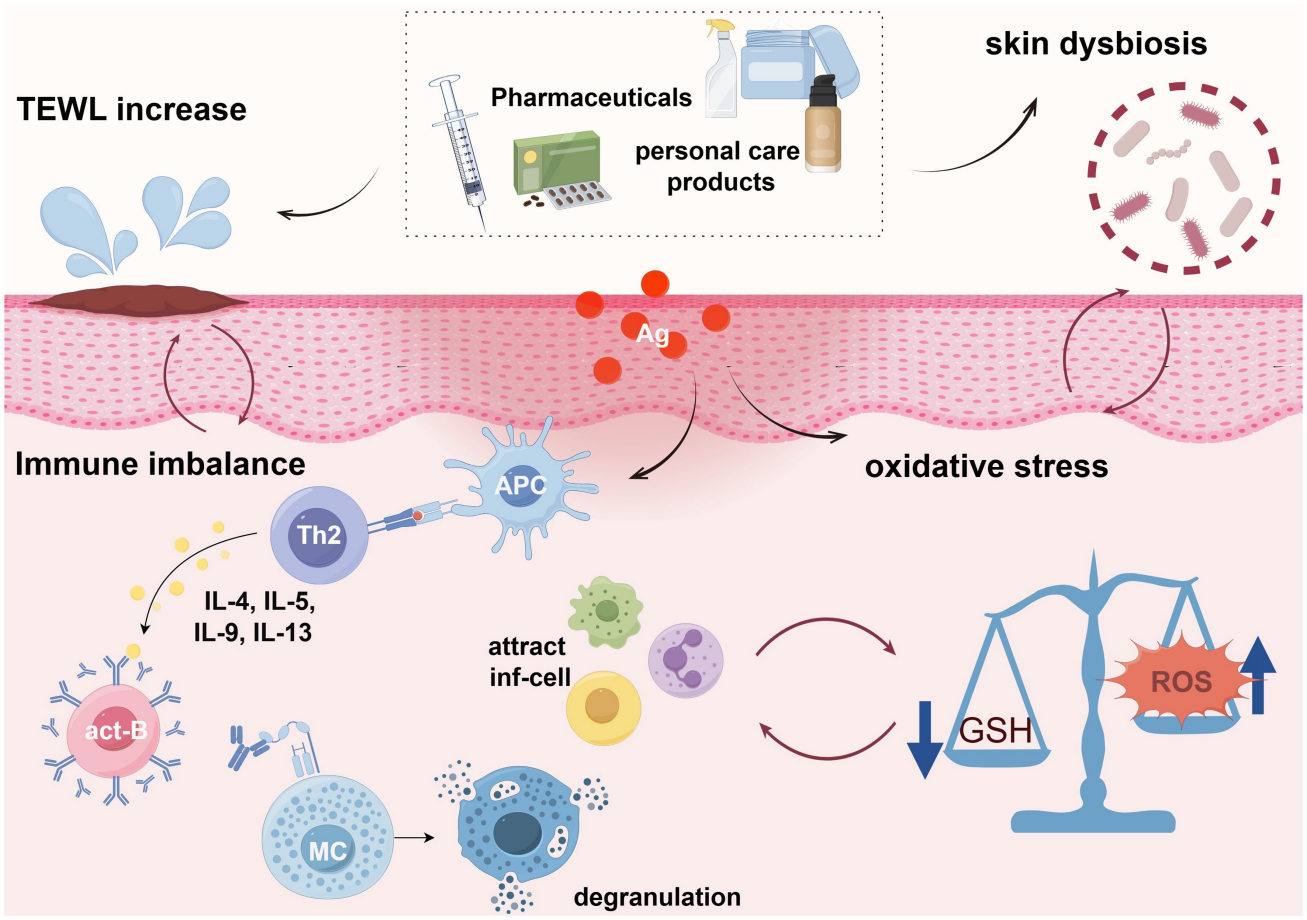
Possible mechanisms of PPCPs leading to AD (By Figdraw.). APC, Antigen-presenting cells; Th2, helper T lymphocytes type 2; act-B, Activated B cells; MC, mast cell; inf-cell, Inflammatory cells; GSH, glutathione; Ag, antigen.

There is no consensus on the specific mechanisms of PPCPs-induced AD. Th2 and Th1/Th17 immune imbalance are recognized as the primary pathological mechanisms of AD (84). In addition to disrupting immune responses, PPCPs also contribute to skin barrier impairment, increased oxidative stress, and microbial dysbiosis (Figure 3).

Most of the existing research is based on mice. In animal experiments, AD models are established using known conventional modeling methodologies by using ovalbumin (OVA), trimellitic anhydride (TMA), fluorescein isothiocyanate (FITC), calcipotriol (MC903) and mite. Researchers often use a certain type of PPCPs to expose mice before or during modeling (with a total exposure duration of 1 month) in order to study the possible pathogenic pathways. The interference of modeling drugs in determining the pathogenicity of PPCPs is inevitable. There is no research that only uses PPCPs for animal exposure studies nowadays. Cells are also reliable research subjects, as they are not affected by other modeling drugs. Two of these studies explored possible pathways, which greatly helps to understand the pathogenic mechanism of PPCPs. Another part of the research is carried out on volunteers, which uses omics as a means of studying specific pathogenic mechanisms usually.

### 4.1 Immune imbalance

Immune imbalance is the most prominent feature of AD. Multiple pollutants have been demonstrated to induce an imbalance in Th2 immunity, which plays a central role in driving AD pathogenesis. The secretion of large amounts of Th2 cytokines prompts B cells to produce large amounts of immunoglobulin (Ig) E and stimulates the release of histamine, cytokines, and chemokines from mast cells, recruiting inflammatory cells to infiltrate the skin (85). Subsequently, Th17 immunity further exacerbates AD and facilitates progression of the disease to asthma, food allergies, etc. (86, 87).

The activation of other innate immune cells is also contributing factors. VOC was shown to stimulate the release of *β*-hexosaminase in bone marrow-derived macrophages and stimulate degranulation of mast cells (88). Additionally, low doses of 4-nonylphenol (NP), 4-tert-octylphenol (OP) and 4-tert-butylphenol (BP)-induced monocyte chemoattractant protein (MCP)-3 and Macrophage inflammatory protein (MIP)-1α can act as chemotactic agents to promote eosinophil chemotaxis in allergic diseases (89). Similar effects were observed of oral administration of DINP and DEHP (90). DINP dose-dependently induced high expression of thymic stromal lymphopoietin (TSLP) and IL-33 in skin keratinocytes, along with activation of epidermal Langerhans cells and dendritic cells, peaking at 60 mg/kg (91).

At the molecular level, the pro-AD effects of PPCPs involve genotypic alterations and signaling pathways. Nuclear factor kappa-B (NF-κB), a typical pro-inflammatory pathway, has been implicated in this process. Blocking NF-κB effectively alleviated symptoms of DINP+OVA-induced AD in mice (91). In addition, Interleukin (IL)-13 gene polymorphisms, in combination with ABX, were associated with an increased risk of AD (*P*_trend_ = 0.06). A more pronounced dose–response relationship was observed in children carrying the IL-13 (rs20541) GA + AA genotype, analyzed as 1-year-old children with AD (31). Epigenetic changes are also important pathogenic factors that cannot be ignored. Two studies have shown that exposure to pollutants can lead to differential expression of genes primarily through low methylation. Janus tyrosine kinase-signal transducer and activator of transcription (JAK–STAT) and phosphatidylinositol 3 kinase-protein kinase B (PI3K-AKT) are the main pathways affected, mediating inflammation and disruption of the epidermal barrier (92, 93).

### 4.2 Microbial dysbiosis

The human microbiome has emerged as a prominent area of research. Microbial communities form complex networks of molecular interactions that influence not only the composition and state of microorganisms within the community but also the state of the host (94, 95). It is of high priority to explore the association between the microbiome and human health and disease. AD has been proven to be associated with abnormal colonization of *Staphylococcus aureus* (*S. aureus*) in the skin. Active *S. aureus* in the stratum corneum and its secreted enzymes and toxins contribute to skin barrier disruption and impaired immune response in AD patients (96, 97). Alterations in the proportion and function of many other strains have also been observed in AD (98).

The skin microbiome is an important component of the skin barrier, and topical exposure to PPCPs may alter the skin microbial balance, thereby increasing the risk of AD. Castillo et al. (99) isolated *Roseomonas mucosa* and *Staphylococcus* spp. from human body and conducted exposure analyses using various PPCPs combinations. Their findings indicated that most of the PPCPs included in the study more strongly inhibited the health-promoting *Roseomonas mucosa* than the disease-promoting *Staphylococcus* spp. Benzyl alcohol, methyldibromo glutaronitrile and glutaraldehyde were the top three PPCPs predicted to have negative effects on microbiome balance. The irrational use of PPCPs has a potential role in dermal microbial ecological dysregulation.

Gut microbial homeostasis is essential for maintaining normal gut immunity and its disruption has been shown to exacerbate AD. ABX can alter specific gut microbiota, affect gut metabolites, disrupt the gut immune system (such as Peyer’s patches), and thus affect the body’s immunity, leading to Th2 dominance (69). After 2 weeks of oral antibiotic cocktail, mice with dysregulated gut microbiota were more likely to be sensitized by OVA and thus showed AD symptoms including increased transepidermal water loss (TEWL) and an intensified inflammatory response. The gut metabolite short-chain fatty acids (SCFAs) may play a major role in maintaining gut immune homeostasis. SCFAs act as modulator of intestinal epithelial cells to support immune function (100). Antibiotic-induced reductions in SCFAs disrupt the balance of intestinal Th17/Treg cells, which in turn affects type 3 natural lymphocytes in the intestinal mucosa and influences the immune status of the organism (101). Study also found that treatment with Azithromycin(AZI), a broad-spectrum antibiotic, at a concentration of 25 mg/kg for 5 days reduced the levels of SCFA-producing intestinal bacteria, including *Alistipes*, *Clostridiales_unclassified* and *Butyricicoccus* and showed a significant negative correlation with AD lesions (102). Population-based studies further confirmed that ABX reduce the relative abundance of probiotics and increase the relative abundance of harmful bacteria, which is thought to be associated with the induction of Th2 immune responses (26, 31).

### 4.3 Barrier impairment

Skin is the first line of defense against external aggressions, making it a critical gateway for pollutant invasion into the human body. A cohort study in Korea (103) showed that high levels of four high-molecular-weight phthalates (*p* = 0.033) and three low-molecular-weight phthalates (*p* = 0.009) led to skin barrier dysfunction in 448 schoolchildren aged 10–12 years. Zhao et al. (104) study also showed that fluoride can interfere with cytoskeleton generation and formation of keratinocytes, making it difficult for skin cells to differentiate and develop normally during the embryonic stage, thereby increasing susceptibility to congenital skin diseases such as AD.

Skin keratinocytes play a critical role in maintaining the skin’s defenses. The secretion of TSLP by epidermal keratinocytes acts as a spearhead of the inflammatory response in AD (105). Several chemicals have been shown to induce the overproduction of TSLP and synergistically shifts the immune system toward Th2 and Th17 immunity by IL-33 (106). It was shown that chloroform-induced phosphorylation of extracellular regulated protein kinases (ERK) and c-Jun N-terminal kinase (JNK) mediated the expression of early growth response (Egr-1), an important mediator of environmental factor-induced inflammatory diseases, in human keratinocytes, thereby inducing TSLP overexpression (107). Furthermore, NF-κB is another possible pathway. For instance, cosmetic coloring agent Lithol Rubine B (LR-B) enhances PMA-induced degradation and phosphorylation of inhibitor kappa B alpha (IκBα) (105).

Abnormalities in the stratum corneum (SC) increase TEWL, leading to skin barrier dysfunction. A German case–control study (108) showed that exposure to 30% n-propanol increased skin erythema and TEWL while reducing natural moisturizing factor (NMF) levels. Similarly, a study by Li et al. (109) demonstrated that a certain ingredient in a skincare product led to abnormalities in epidermal function in mice, including elevated TEWL and surface pH, as well as reduced SC water content. In addition, skin exposure to 5-chloro-2-methylisothiazol-3(2H)-one and 2-methylisothiazol-3(2H)-one (CMIT/MIT) leads to elevated TEWL, scaling, abrasion and keratinocyte damage (110). Filaggrin (FLG) is a key factor in maintaining epidermal moisturization. Gene–environment interactions between FLG variants and pollutant exposure have been reported to increase susceptibility to AD. For instance, the FLG P478S TT genotype is associated with increased AD incidence in children exposed to high levels of MBP (OR ¼ 4.74, 95% CI 1.45–15.5) and MBzP (OR ¼ 3.46, 95% CI 1.03–11.58). This may be due to variations in the FLG gene increases skin permeability (111). Toluene exposure has also been found to decrease FLG mRNA and protein levels. Additionally, NMF, a breakdown product of polysilk proteins composed of pyrrolidone carboxylic acid and uronic acid, was reduced by toluene treatment. This effect results from phosphorylation of ERK and activation of STAT3 in keratin-forming cells (112).

### 4.4 Oxidative stress

Oxidative stress has been found to contribute to the development of AD. During inflammation, excess reactive oxygen species (ROS) accumulate at the lesion site (113, 114), causing severe oxidative damage, including membrane lipid peroxidation and damage to DNA and proteins (115). Additionally, ROS are involved in signaling pathways such as NF-κB and p38 mitogen-activated protein kinase (p38-MAPK), inducing aberrant T-cell differentiation and macrophage polarization (116, 117). This malignant interaction exacerbates the progression of AD.

Some PPCPs have been shown to induce oxidative stress. Glutathione-S-transferase (GST) is an antioxidant substance. Study has shown that children with GSTT1-deficient genotype (OR 3.45, 95%CI 1.26–9.99) or GSTM1-deficient genotype (OR 2.92, 95%CI 1.12–7.91) are at a higher risk of developing AD when exposed to high levels of PFOA. It is hypothesized that PFOA induce ROS production, and children with GSTT1- or GSTM1-deficient genotypes, due to the loss of enzyme activity, may be more susceptible to oxidative stress (73). Additionally, high concentrations of p-aminobenzoic acid have been found to differentially interfere with three major metabolic pathways (amino acid, carbohydrate, and lipid metabolism) (69). The author noted that changes in carbohydrate and amino acid levels are thought to induce ROS production. Reduced levels of palmitic acid and 2-palmitoylglycerol, along with increased levels of pyridinecarboxylic acid interfered to varying degrees with the peroxisome proliferator-activated receptor pathway and the aryl hydrocarbon receptor signaling pathway. These disruptions subsequently diminished the body’s antioxidant capacity and interfered with immune homeostasis (69).

PPCPs significantly contribute to the deterioration of AD by disrupting the skin barrier through the mechanisms described above. However, the exact mechanisms of each pollutant and its detailed correlation with AD have not been fully elucidated, and different mechanisms may overlap. Table 4 provides a summary of the aforementioned mechanistic studies.

**Table 4:** Exploration of the mechanism related to PPCPs and AD.

Pollutant	Object	Result	Exposure pathway	Dose	Duration	Irritant	Result	Ref.
Antibiotic mixture	BALB/c mice (female) (3 w)	+	take orally	ampicillin (1 g/L), vancomycin (500 mg/L), ciprofloxacin (200 mg/L), imipenem (250 mg/L), metronidazole (1 g/L).	2 w	OVA	Rising TEWL, IgE, and IL-4 caused skin lesions. Reduced SCFA levels boosted intestinal CD4 + IL-17+, CD4 + FOXP3 + Tregs, and group 3 ILCs.	(101)
Azithromycin	BALB/c mice (male) (3–6 w)	**+**	gavage	25 mg/kg	5 d	TMA	Skin swelling and itching sensation. Th2/Th17 immune responses were enhanced. Depleted three SCFA producing gut bacterial genera (Alistipes, Clostridiales_unclassified, Butyricicoccus).	(102)
Dexamethasone	THP-1 cell	**+**	/	100 nM	overnight	/	Inhibit the expression of aromatase in peripheral monocytes, leading to a decrease in skin elasticity and moisture.	(154)
Diisononyl phthalate	BALB/c mice (male) (4 w)	**+**	gavage	0.15–60 mg/kg	18 d	OVA	Thickened epidermis, heightened TSLP/IL-33 secretion by keratinocytes, activated Langerhans/dendritic cells, elevated Th2/Th17 cytokines (boosting IgE), reduced IFN-γ. NF-κB implicated.	(91)
DINP	NC/Nga mice (male) (7 w)	**+**	take orally	6.6-2625 mg/animal	4 w	mite	Exacerbate AD-like skin damage, and increased expression of MIP-1a. Increase inflammatory cell infiltration, degranulation of mast cells, and local expression of MIP-1a.	(90)
DEHP		**+**		8.3-3325 mg/animal				
Dibutyl phthalate	BALB/c mice (male) (5–6 w)	**+**	skin exposure	4.0–40.0 mg/kg	40 d	FITC	Elevated levels of Th2/Th17 cytokines. Enhanced eosinophil accumulation, mast cell degranulation, and TSLP expression.	(155)
VOC (Toluene)	3D skin model	**+**	/	50 nM	/	/	Reduced filaggrin expression, lowering NMF and its components via ERK/STAT3 pathway activation.	(112)
CMIT/MIT	BALB/c mice (male) (5 w)	**+**	skin exposure	0.1875 mg/kg	3 w	OVA	Elevated TEWL, erythema, scales, abrasions and skin inflammation. Elevated serum IgE, dermal inflammatory cells and skin Th2 responses. Th17 cytokines/cells emerged later.	(110)
BZK	NC/Nga mice (male) (10 w)	**+**	skin exposure	0.2%	18 d	mite	Elevated subcutaneous IL-1β/IL-33/IL-18/MIP-1α, serum IgE, inflammatory infiltrates, and TNF-α/RANTES rise. BZK was most pathogenic (PVP-I weaker, CHG neutral).	(106)
PVP-I		**+**		10%				
CHG		−		0.5%				
NP	NC/Nga mice (male) (7 w)	**+**	intraperitoneal injection	1.75–1750 μg/animal	16 d	mite	Enhanced Th2 immunity. IL-4, IL-6, TSLP, MCP-3, MIP-1 α, total IgE and antigen-specific IgG1 all increased.	(89)
OP		**+**		2.625–2,625 μg/animal				
BP		**+**		10.5–10,500 μg/animal				
PCPs	mice (male) (6–8 w)	**+**	skin exposure	/	2 d	MC903	Most skincare products promote the growth of disease-related bacterial strains more than health-related isolates.	(99)
Chloroform	HaCaT cell	**+**	/	250 μM	2 h	/	Chloroform dose-dependently boosts TSLP in keratinocytes via ERK/JNK-mediated Egr-1 activation.	(107)
AcA + AcA	human (20–65 Y)	−	skin exposure	/	4 d	/	Exposure to SLS has a more significant impact on NMF. In AD patients, any exposure can increase the TEWL. In healthy controls, only SLS caused serious consequences.	(156)
AcA + SLS		**+**		/				
SLS + AcA		**+**		/				
SLS + SLS		**+**		/				
LR-B (LR-BCA)	BALB/c mice	**+**	skin exposure	1%	5d	MC903	LR-B/LR-BCA boost TSLP, triggering Th2 response via NF-κB activation (not AP-1) in keratinocytes.	(105)
PFOS	hESC H1 cell	+	/	10 μM	10 d	/	Transformed non-neuroectodermal cells from spindle to pebble-shaped, altered markers (KRT16, etc.). Disrupted keratinocyte genes (KRT6A, etc.), signaling pathways (TGF-β, NOTCH, etc.), and cytoskeletal genes, inhibiting cilia-mediated intercellular signaling.	(104)
VOC (1,3-Butadiene and toluene)	BMMC	+	/	2.2/4.4 ppm; 0.1/0.01 ppm	1 h	compound 48/80	Stimulated the release of β- hexosaminidase in BMSC and stimulates degranulation of mast cells.	(88)
BPA	human (≥18 Y)	+	/	/	/	/	BPA/BPF/BPS exposure altered methylation of AD genes, with key genes (IL4, STAT3, etc.) hypomethylated, impacting JAK–STAT/PI3-AKT pathways.	(92)
BPF				/				
BPS				/				
VOC	human (mother and baby)	+	/	/	/	/	Causes low-methylation-driven differential AD gene expression. Disrupting offspring IL31RA (JAK–STAT pathway), CCL20 (maintain mucosal lymphoid tissue function), and maternal HRH1 (related with skin barrier recovery).	(93)

w: weeks; d: days; min: minutes; +: Increased risk of AD; −: not related to increased risk of AD onset; BZK: benzalkonium chloride; PVP-I: povidone-iodine; CHG: chlorhexidine gluconate; AcA: 2.0% acetic acid; SLS: 0.5% sodium lauryl sulfate; LR-BCA: LR-B’s calcium salt; BMMC: mast cells derived from mouse bone marrow.

## 5 Factors interfering with the results

### 5.1 Race and socio-economic conditions

The prevalence and symptoms of AD are more pronounced in Asians and Africans, but the reasons remain unclear (118, 119). Most epidemiologic investigations focus on Europe and Southeast Asia, with limited data from Africa and other parts of Asia. However, only a few studies specified that their participants were from the same race or corrected the analysis for ethnicity. Socio-economic factors also influence AD outcomes, as poorer living conditions and social pressure can lead to abnormal AD progression (120). Quantifying this influence is challenging, and studies often rely on parental income and education to adjust for socio-economic impacts, which is insufficient. Additionally, poorer socio-economic conditions are often linked to inadequate medical care, thus some studies rely on questionnaires rather than doctor diagnosis increase the uncertainty of results.

### 5.2 Gender and age of participants

Studies suggest that the association between pollutant exposure and AD varies by gender and age. Gender differences in pollutant metabolism necessitate gender-specific analyses. Some PPCPs have estrogen mimicking effect, which has been proved to increase the incidence rate of allergic diseases (121). Endocrine-immune axis interactions have been widely discussed (122). Early life is a critical period for AD patient to establish immune tolerance (123), leading to most studies focusing on young children. However, exposure in adolescents and special populations (e.g., immunodeficient populations, pregnant women, etc.) also deserves attention due to the rapid changes in its endocrine homeostasis. In addition, late-onset AD, despite most cases developing before age two, also deserves separate study due to differing causative factors (124).

### 5.3 Subtypes of AD and IgE levels

AD can be categorized into different subtypes based on age, disease duration and IgE levels, and its pathogenic mechanisms are significantly different (125). Precision therapy targeting these mechanisms could promote personalized AD treatment (126, 127). Some investigations found unexplained correlations among AD, IgE and contaminant concentrations, but only one study clearly identified differences between exotropic and endotropic AD when exposed to pollutants, suggesting current research is insufficient to meet the demand for precision medicine.

### 5.4 Limitations of research types and samples

Research on PPCPs and AD is still dominated by cross-sectional studies or retrospective cohort studies. Urine and blood are the most commonly used sample because of their non-invasiveness. However, single time-point detection results may not reflect long-term exposure due to varying pollutant half-lives (128). Furthermore, urinary or blood residues of PPCPs metabolites do not account for different exposure routes. Even at the same exposure dose, different exposure routes lead to varied outcomes, probably because they activate different types of immunological responses (129). This partly explains inconsistent findings across studies. Most pollutant exposure is assumed to be transdermal, but oral exposure, especially in children due to licking, should not be overlooked. PPCPs contamination in water sources also requires further analysis to establish appropriate standards (130).

Attention should also be paid to the issue of sample size in research. Reasonable determination of sample size is crucial in research design. Some studies have shown that the risk indicators associated with certain pollutants and the onset of AD are close to 1, indicating a weak effect. From a statistical perspective, the validation of such weak effects requires a higher sample size. Only with a sufficiently large sample size can random error interference be eliminated. However, about a quarter of existing studies have not met the minimum sample size required for statistics. For studies with statistical significance at a critical value (such as a *p*-value close to 0.05), this issue may constitute false negatives or false positives in the conclusions, ultimately affecting the scientific validity and credibility of the research findings.

Another noteworthy aspect is that, as an important component of the human genome, exploring the relationship between the microbiome and human health and disease is of paramount importance. Existing research has shown that the homeostasis of skin microbiota can affect the onset of AD, and abnormal colonization of pathogenic bacteria such as *Staphylococcus aureus* is a recognized pathogenic factor for AD. However, few studies have mentioned the impact of pollutants on the homeostasis of skin microbiota. More research on the skin microbiota should be considered.

### 5.5 Contradictory animal and human research results

The short exposure period and high pollutant dose of most animal experiments do not reflect real-world long-term, low-dose exposures. And the number of relevant animal experiments is limited compared to the hot pollutants in epidemiological studies. In addition, epidemiologic studies revealed links between single PPCPs exposure and AD, while animal studies often require allergens or adjuvants to establish AD models. This may be because most studies only examined one PPCPs metabolite, ignoring potential interactions with others. The synergistic and antagonistic effects of pathogenic and protective PPCPs are worthy of further research.

## 6 Suggestions and prospects

AD patients exposed to widespread PPCPs should be cautious about care product choices. Choosing care products that are clearly labeled as “Fragrance Free” and have a simple ingredient list can effectively reduce contact with phthalates (plasticizers and fixatives) and parabens (preservatives). In addition, it is better to prioritize products packaged in metal or glass materials, as BPA may be present in packaging materials such as plastic bottles. VOC may be present in certain volatile silicone oils or alcohols in moisturizers, so avoid using products that have a strong odor or evaporate quickly. For young children, fluoride free toothpaste should be given priority when choosing toothpaste. Factors like cleansing product pH and water hardness also affect AD prevalence (131, 132). There is a positive correlation between hard water exposure and AD. Consequently, using a water softener to improve water quality may help prevent the further progression of the disease. Additionally, the skin pH level at AD-affected sites tends to increase. For this reason, selecting weakly acidic, moisturizing cleansers can aid in alleviating AD symptoms (133). The living environment is also an important factor affecting the exposure of PPCPs. Reducing dust, ventilating well, and picking safe hygiene products help are the right choices (134). Secondly, physicians should tailor medication plans by assessing infection severity. The prevention of AD infection should emphasize restoring the skin barrier and mitigating type 2 inflammation, rather than relying on antibiotics. Acute phase response markers, such as C-reactive protein and erythrocyte sedimentation rate, may help determine whether a patient needs antibiotics (135). Even when antibiotics have to be used, targeted medication should be administered based on the severity of infection and the type of pathogen. Finally, macro-control by the government is decisive. Strict supervision over the amount of PPCPs is required. Meanwhile, waterborne testing of pollutants with efficient removal methods should be strengthened.

In summary, AD patients’ exposure to PPCPs is an issue that cannot be prevented in the near future. Epidemiologic evidence indicates that PPCPs are, in fact, a negative factor in the development and course of AD. To properly quantify risk, further study is required given the reality of numerous routes of ingestion and cumulative PPCPs exposures. Future research could explore the specific mechanisms of PPCPs in AD pathogenesis at the molecular level. For instance, certain PPCPs may interact with specific receptors or signaling pathways linked to AD development. Clarifying the interactions of pollutants-target receptors-downstream pathways, alongside identifying the true pathogenic moieties of the pollutants, will be crucial. Leveraging modern bioinformatics and molecular biology techniques could further facilitate the development of more targeted therapeutic agents for AD.
