# Supplementary material for: A review of pharmaceutical and personal care products and atopic dermatitis based on epidemiological and basic research findings
Source: Front Public Health. 2025 Sep 25;13:1642873. doi: 10.3389/fpubh.2025.1642873 (PMC12507775; doi:10.3389/fpubh.2025.1642873)
Supplement: Supplementary file 1 [file Table_1.DOCX]

Supplementary Material

**Supplementary Table 1** The correlation between other drugs exposure and the risk of AD

| **Pollutant** | **Method** | **Country/Region** | **Population** | **Exposure period** | **Sample size** | **Correlation** | **Risk indicator;**  **95%CI** | **P-value** | **reference** |
| --- | --- | --- | --- | --- | --- | --- | --- | --- | --- |
| Laxative | cohort study | Southampton | 6 M | early intrauterine period | 3158 | - | O 0.46; 0.14-1.49 | 0.20 | (1) |
|  |  |  | 12 M |  |  | - | 0.99; 0.42-2.33 | 0.98 |  |
|  |  |  | 6 M | late intrauterine period |  | - | 0.91; 0.39-2.14 | 0.83 |  |
|  |  |  | 12 M |  |  | - | 1.25; 0.59-2.66 | 0.56 |  |
| H2RA | retrospective cohort study | Bethesda | 4.6 Y (Me) | 0-6 M | 792130 | + | H 1.12; 1.09-1.14 | / | (2) |
| PPI |  |  |  |  |  | + | 1.12; 1.07-1.17 | / |  |
| ASM | cohort study | Korea | 6 M-13 Y | intrauterine period | 3904536 | + | H 1.02; 1.01-1.02 | / | (3) |
|  |  |  |  | 0-6 M | 3506031 | + | 1.05; 1.02-1.08 | / |  |
| ASM | cohort study | Netherlands | 0-8 Y | intrauterine period | 33536 | + | H 1.32; 1.06-1.64 | / | (4) |
| H2RA |  |  |  |  |  | - | 1.42; 0.98-2.06 | / |  |
| PPI |  |  |  |  |  | - | 1.24; 0.94-1.65 | / |  |
| ASM | retrospective cohort study | Taiwan | ≥18 Y | within one year or continuously more than 90 D | 109980 | + | H 1.52; 1.40-1.64 | *P*< 0.001 | (5) |
| H2RA | retrospective cohort study | Japan | 1-10 Y | 0-1 Y | 85954 | + | H 1.12; 0.75-1.67 | / | (6) |
| Tacrolimus | retrospective cohort study | Japan | 2/8 Y ^a.^ | intrauterine period | 85 | - | O 0.58; 0.06-4.50 | 0.60 | (7) |
| Acetaminophen | nested case-control study | Taiwan | 0-10 Y | intrauterine period | 7087 | + | O 1.12; 1.00-1.26 | ＜0.05 | (8) |
| Acetaminophen | multi-central cross-sectional study | 22 countries | 6-7 Y | 0-1 Y | 120799 | + | O 1.28; 1.21-1.36 | / | (9) |
|  |  |  |  | current | 120799 | + | 1.45; 1.37-1.54 | / |  |
|  |  |  | 13-14 Y |  | 224436 | + | 1.57; 1.51-1.63 | / |  |
| Acetaminophen | retrospective cohort study | Taiwan | 0-10 Y | intrauterine period | 1288343 | + | H 1.11; 1.10-1.12 | / | (10) |
| Acetaminophen | retrospective cohort study | Japan | 1-10 Y | 0-1 Y | 85954 | - | H 1.02; 0.94-1.11 | / | (6) |
| Acetaminophen | cross-sectional study | Kosovo | 13-14 Y ^b^ | in the last 12 M (＞1 times/year) | 6682 | - | O 1.50; 0.98-2.30 | / | (11) |
|  |  |  |  | in the last 12 M (＞1 times/month) |  | + | 2.75; 1.81-4.17 | / |  |
|  |  |  | 13-14 Y | in the last 12 M (＞1 times/year) |  | - | 1.16; 0.83-1.62 | / |  |
|  |  |  |  | in the last 12 M (＞1 times/month) |  | + | 1.44; 1.02-2.03 | / |  |
| Acetaminophen | cross-sectional study | Saudi Arabia | 6-7 Y | intrauterine period (＞1 times/month) | 3614 | - | O 1.15; 0.62-2.14 | 0.663 | (12) |
|  |  |  |  | 0-1 Y |  | - | 1.36; 0.68-2.75 | 0.388 |  |
|  |  |  |  | in the last 12 M (＞1 times/month) |  | - | 1.42; 0.58 -3.4 | 0.441 |  |
|  |  |  |  | in the last 12 M (＞1 times/year) |  | - | 1.59; 0.65-3.89 | 0.306 |  |
|  |  |  | 13-14 Y | in the last 12 M (＞1 times/month) | 4086 | + | 2.43; 1.31-4.49 | 0.005 |  |
|  |  |  |  | in the last 12 M (＞1 times/year) |  | - | 1.76; 0.94-3.30 | 0.076 |  |
| THB | retrospective cohort study | Japan | 1-10 Y | 0-1 Y | 85954 | - | H 1.08; 0.96-1.21 | / | (6) |
| Ambroxol | retrospective cohort study | Japan | 1-10 Y | 0-1 Y | 85954 | Re | H 0.76; 0.60-0.87 | / | (6) |
| Carbocysteine | retrospective cohort study | Japan | 1-10 Y | 0-1 Y | 85954 | - | H 1.03; 0.93-1.13 | / | (6) |
| LTRA | retrospective cohort study | Japan | 1-10 Y | 0-1 Y | 85954 | - | H 1.09; 0.94-1.28 | / | (6) |
| H1RA | retrospective cohort study | Japan | 1-10 Y | 0-1 Y | 85954 | + | H 1.23; 1.12-1.36 | / | (6) |
| GA | retrospective cohort study | Taiwan | 0-1 Y | 0-1 Y | 32742 | Re | H 0.60; 0.56-0.64 | / | (13) |
| GA | cohort study | Korea | 0-18 Y | 0-18 Y | 46086 | - | H 1.03; 0.87-1.23 | 0.701 | (14) |
| Palivizumab | cohort study | Denmark  &Sweden | 0-4 Y | 0-2 Y | 1351265 | - | H 0.92; 0.64-1.34 | / | (15) |
|  |  |  | premature infant |  | 60461 | - | 1.33; 0.70-2.51 | / |  |

Y: years old; M: months old; D: days old; H: Hazard Ratio; O: Odds Ratio; R: Relative Risk; +: Increased risk of AD; Re: Reduce the risk of AD; -: not related to increased risk of AD onset;Me: median;THB‌‌: Tipepidine hibenzate; H1RA: Histamine H1 receptor antagonist; LTRA: Leukotriene receptor antagonist.

a.: The first number is the median age of the control group, and the second number is the median age of the exposed rent. b.: This line shows the relationship between current AD and pollutants, while the following line shows the previous relationship between AD and pollutants.

**Supplementary Table 2** The correlation between phthalates exposure and the risk of AD (Negative Result)

| **Pollutant** | **Concentration** ^a.^ | **Method** | **Country/Region** | **Population** | **Sample** | **Sample size** | **Correlation** | **Risk indicator;**  **95%CI** | **P-value** | **reference** |
| --- | --- | --- | --- | --- | --- | --- | --- | --- | --- | --- |
| ∑DEHP | 0.49 (GM; μg/g Cr) | case-control study | Seoul | 3-6 Y | urine | 448 | - | O 1.05; 0.96-1.15 | / |  |
| ∑DEHP | 0.109 (GM) | cross-sectional study | Korea | 3-5 Y | urine | 571 | - | odds 1.56; 0.87-2.80 | / | (17) |
| MEHHP | ^b.^ |  |  |  |  |  | - | 1.57; 0.91-2.70 | / |  |
| MEOHP |  |  |  |  |  |  | - | 1.68; 0.94-3.00 | / |  |
| MECPP |  |  |  |  |  |  | - | 1.30; 0.73-2.31 | / |  |
| MBzP | 0.00312 |  |  |  |  |  | - | 1.00; 0.77-1.30 | / |  |
| MnBP | 0.0472 |  |  |  |  |  | - | 1.08; 0.85-1.39 | / |  |
| MCOP | 0.00162 |  |  |  |  |  | - | 1.19; 0.71-2.02 | / |  |
| MCNP | 0.000490 |  |  |  |  |  | - | 1.14; 0.71-1.84 | / |  |
| MCPP | 0.00180 |  |  |  |  |  | - | 1.21; 0.74-2.00 | / |  |
| ∑DEHP | 0.0940 |  |  | 6-11 Y |  | 887 | - | 1.25; 0.83-1.89 | / |  |
| MEHHP | ^b.^ |  |  |  |  |  | - | 1.10; 0.64-1.88 | / |  |
| MEOHP |  |  |  |  |  |  | - | 1.25; 0.87-1.77 | / |  |
| MECPP |  |  |  |  |  |  | - | 1.10; 0.70-1.73 | / |  |
| MBzP | 0.00280 |  |  |  |  |  | - | 1.12; 0.90-1.38 | / |  |
| MnBP | 0.0432 |  |  |  |  |  | - | 1.25; 0.79-1.99 | / |  |
| MCOP | 0.00224 |  |  |  |  |  | - | 1.23; 0.87-1.74 | / |  |
| MCNP | 0.000533 |  |  |  |  |  | - | 1.06; 0.68-1.64 | / |  |
| MCPP | 0.00156 |  |  |  |  |  | - | 0.97; 0.69-1.37 | / |  |
| DiBP | 1.51 (Me; μg/kgBW/day)c^.^ | cross-sectional study | Sapporo | 6-12 Y | urine | 184 | - | O 0.85; 0.68-1.05 | 0.132 | (18) |
| DnBP | 0.07 |  |  |  |  |  | - | 0.98; 0.62-1.54 | 0.921 |  |
| BBzP | 0.48 |  |  |  |  |  | - | 0.99; 0.74-1.33 | 0.936 |  |
| MEHP | 9.80 |  |  |  |  |  | - | 1.03; 0.81-1.31 | 0.826 |  |
| MEOHP | 8.61 |  |  |  |  |  | - | 1.16; 0.73-1.84 | 0.520 |  |
| MECPP | 3.90 |  |  |  |  |  | - | 0.75; 0.48-1.18 | 0.212 |  |
| DEHP | 7.68 |  |  |  |  |  | - | 1.02; 0.69-1.52 | 0.921 |  |
| DEP | 24.61 (Me; μg/g Cr) | multi-central prospective cohort study | Łó d ź Region | 0-2 Y | urine | 144 | - | O 1.09; 0.27-4.38 | / | (19) |
| DiBP | 13.46 |  |  |  |  |  | - | 0.84; 0.43-1.67 | / |  |
| DnBP | 14.15 |  |  |  |  |  | - | 0.83; 0.24-2.89 | / |  |
| MnBP | 5.19 |  |  |  |  |  | - | 0.67; 0.25-1.84 | / |  |
| 3OH-MnBP | 5.42 |  |  |  |  |  | - | 1.31; 0.29-5.85 | / |  |
| BBzP | 0.05 |  |  |  |  |  | - | 3.39; 0.77-14.80 | / |  |
| DEHP | 8.10 |  |  |  |  |  | - | 0.36; 0.08-1.51 | / |  |
| MEHP | 0.24 |  |  |  |  |  | - | 0.47; 0.13-1.75 | / |  |
| MEHHP | 3.59 |  |  |  |  |  | - | 0.70; 0.29-1.70 | / |  |
| MEOHP | 1.56 |  |  |  |  |  | - | 0.46; 0.14-1.59 | / |  |
| DiNP | 1.09 |  |  |  |  |  | - | 0.59; 0.13-2.65 | / |  |
| 7OH-MiNP | 1.09 |  |  |  |  |  | - | 0.59; 0.13-2.65 | / |  |
| 7oxo-MiNP | 0.36 |  |  |  |  |  | - | 5.46; 0.61-48.93 | / |  |

Y: years old; H: Hazard Ratio; O: Odds Ratio; Me: median; GM: geometric mean; DEHP: Di(2-ethylhexyl) phthalate; DINP: di-isononyl phthalate; DnBP: di-n-butyl phthalate; MEHHP: mono(2-ethyl-5-hydroxyhexyl)phthalate; 7OH-MiNP: mono(4-methyl-7-hydroxyoctyl)phthalate; 7oxo-MiNP: mono(4-methyl-7-oxo-octyl)phthalate; BBzP: butyl benzyl phthalate; DEP: di-ethyl phthalate; DiBP: di-isobutyl phthalate; MBzP: monobenzyl phthalate; MCNP: monocarboxyononyl phthalate; MCOP: monocarboxyoctyl phthalate; MCPP: mono(3-carboxypropyl)phthalate; MECCP: mono(2-ethyl-5-carboxypentyl)phthalate; MEHP: mono-(2-ethylhexyl)phthalate; MEOHP: mono(2-ethyl-5-oxohexyl)phthalate; MnBP: mono-n-butyl phthalate.

**Supplementary Table 3** The correlation between fluoride exposure and the risk of AD

| **Pollutant** | **Concentration^a.^** | **Method** | **Country/Region** | **Population** | **Sample** | **Sample size** | **Correlation** | **Risk indicator;**  **95%CI** | **P-value** | **reference** |
| --- | --- | --- | --- | --- | --- | --- | --- | --- | --- | --- |
| fluoride | 710000 (IQR) | cohort study | Northern Sweden | 1 Y | urine (intrauterine period ≤ 29 W) | 467 | - | O 1.40; 0.91-2.15 | / | (20) |
|  | 40000 |  |  |  | urine (0-4 M) | 321 | - | 1.43; 0.97-2.12 | / |  |
| PFOS | / | cohort study | Denmark | 6 Y | plasma (intrauterine period 24 W) | 668 | - | O 1.01; 0.96-1.06 | 0.70 | (21) |
|  | / |  |  |  | plasma (0-1 W) | 658 |  | 0.99; 0.93-1.06 | 0.80 |  |
| PFOA | / |  |  |  | plasma (intrauterine period 24 W) | 668 | - | 0.94; 0.74-1.20 | 0.60 |  |
|  | / |  |  |  | plasma (0-1 W) | 658 |  | 0.95; 0.70-1.29 | 0.70 |  |
| PFHpA | 0.13 (IQR) | prospective cohort study | Oslo | 10-16 Y | serum | 378 | - | R 0.95; 0.76-1.20 | 0.69 | (22) |
| PFOA | 1.77 |  |  |  |  |  | - | 0.97; 0.78-1.21 | 0.80 |  |
| PFNA | 0.29 |  |  |  |  |  | - | 0.83; 0.65-1.08 | 0.16 |  |
| PFDA | 0.13 |  |  |  |  |  | - | 0.86; 0.66-1.12 | 0.28 |  |
| PFUnDA | 0.13 |  |  |  |  |  | - | 0.95; 0.75-1.21 | 0.69 |  |
| PFHxS | 0.86 |  |  |  |  |  | Re | 0.79; 0.34-0.99 | 0.04 |  |
| PFHpS | 0.20 |  |  |  |  |  | - | 0.98; 0.83-1.17 | 0.84 |  |
| PFOS | 9.23 |  |  |  |  |  | - | 0.93; 0.73-1.20 | 0.59 |  |
| PFOSA | 0.25 |  |  |  |  |  | - | 1.14; 0.68-1.93 | 0.62 |  |
| PFHxS | 23.00 (Me) | cohort study | Taiwan | 2 Y | cord blood | 839 | - | O 1.37; 0.60-3.18 | *P*＞0.05 | (23) |
| PFOA | 0.65 |  |  |  |  |  | + | 2.58; 1.27-5.32 | *P＜*0.01 |  |
| PFNA | 0.93 |  |  |  |  |  | - | 0.44; 0.18-1.01 | *P*＞0.05 |  |
| PFOS | 3.48 |  |  |  |  |  | - | 1.86; 0.84-4.36 | *P*＞0.05 |  |
| PFUnDA | 0.67 |  |  |  |  |  | - | 0.54; 0.23-1.17 | *P*＞0.05 |  |
| PFDoDA | 0.25 |  |  |  |  |  | - | 0.60; 0.26-1.33 | *P*＞0.05 |  |
| PFUnDA | 0.20 (Me) | cohort study | Norway | 7 Y | plasma (from mother) | 921 | Re | O 0.69; 0.55-0.86 | 0.001 | (24) |
| PFOS | 12.87 |  |  |  |  |  | - | 1.03; 0.80-1.33 | 0.798 |  |
| PFOA | 2.54 |  |  |  |  |  | - | 1.02; 0.77-1.34 | 0.914 |  |
| PFHxS | 0.65 |  |  |  |  |  | - | 1.09; 0.90-1.31 | 0.389 |  |
| PFNA | 0.45 |  |  |  |  |  | - | 0.90; 0.70-1.17 | 0.440 |  |
| PFHpS | 0.15 |  |  |  |  |  | - | 0.95; 0.79-1.15 | 0.608 |  |
| PFOS | 2.48 (Me) | prospective cohort study | Shanghai | 2 Y | cord blood | 687 | - | O 1.23; 0.85–1.76 | 0.33 | (25) |
| PFOA | 6.98 |  |  |  |  |  | - | 1.35; 0.93–1.97 | 0.17 |  |
| PFNA | 0.64 |  |  |  |  |  | - | 1.53; 0.94–2.47 | 0.46 |  |
| PFDA | 0.36 |  |  |  |  |  | - | 1.22; 0.94–1.58 | 0.65 |  |
| PFUA | 0.4 |  |  |  |  |  | - | 1.24; 0.88–1.75 | 0.67 |  |
| PFDoDA | 0.09 |  |  |  |  |  | - | 1.00; 0.72–1.39 | 0.28 |  |
| PFHxS | 0.16 |  |  |  |  |  | - | 1.08; 0.62–1.85 | 0.43 |  |
| PFBS | 0.05 |  |  |  |  |  | - | 1.22; 0.87–1.72 | 0.83 |  |
| PFPA | 0.13 (Me) | longitudinal cohort study | The Czech Republic | adult | serum | 309 | - | O 0.90; 0.53-1.54 | / | (26) |
| PFOA | 1.11 |  |  |  |  |  | Re | 0.58; 0.37-0.90 | / |  |
| PFNA | 0.31 |  |  |  |  |  | - | 0.58; 0.34-1.00 | / |  |
| PFDA | 0.13 |  |  |  |  |  | - | 0.72; 0.46-1.13 | / |  |
| PFUnDA | 0.06 |  |  |  |  |  | - | 0.74; 0.46-1.19 | / |  |
| PFBS | 0.06 |  |  |  |  |  | - | 1.02; 0.61-1.69 | / |  |
| PFHxS | 0.32 |  |  |  |  |  | - | 0.69; 0.40-1.19 | / |  |
| PFHpS | 0.04 |  |  |  |  |  | - | 0.70; 0.49-1.00 | / |  |
| PFOS | 1.93 |  |  |  |  |  | Re | 0.56; 0.32-0.95 | / |  |
| PFOA | 1.96 (the third quartile) | cohort study | Taiwan | 0-5 Y | cord blood | 863 | + | H1.89; 1.10-3.16 | < 0.05 | (27) |
| PFOS | 5.05 |  |  |  |  |  | - | 1.43; 0.82-2.43 | ＞0.05 |  |
| PFOS | 5.6 (mean) | prospective cohort study | Oslo | 0-2 Y | cord blood | 641 | - | O 1.15; 0.88-1.52 | 0.310 | (28) |
|  |  |  |  | 0-10 Y |  |  | - | 0.68; 0.38-1.20 | 0.181 |  |
| PFOA | 1.8 |  |  | 0-2 Y |  |  | - | 1.18; 0.94-1.50 | 0.159 |  |
|  |  |  |  | 0-10 Y |  |  | - | 0.99; 0.59-1.67 | 0.971 |  |
| PFOSA | 0.4 |  |  | 0-2 Y |  |  | - | 1.09; 0.94-1.26 | 0.263 |  |
|  |  |  |  | 0-10 Y |  |  | - | 1.21; 0.85-1.72 | 0.290 |  |
| PFHxS | 0.3 |  |  | 0-2 Y |  |  | - | 1.06; 0.89-1.26 | 0.518 |  |
|  |  |  |  | 0-10 Y |  |  | - | 1.00; 0.67-1.49 | 0.995 |  |
| PFNA | 0.2 |  |  | 0-2 Y |  |  | - | 1.03; 0.85-1.24 | 0.762 |  |
|  |  |  |  | 0-10 Y |  |  | - | 1.12; 0.75-1.68 | 0.586 |  |
| PFUnDA | 0.1 |  |  | 0-2 Y |  |  | - | 1.02; 0.85-1.24 | 0.802 |  |
|  |  |  |  | 0-10 Y |  |  | - | 1.20; 0.79-1.83 | 0.387 |  |

Y: years old; M: months old; H: Hazard Ratio; O: Odds Ratio; R: Relative Risk; +: Increased risk of AD; Re: Reduce the risk of AD; -: not related to increased risk of AD onset; *: associated with worsening symptoms of AD; Me: median; IQR: interquartile range; PFBS: perfluorobutanesulfonic acid; PFDA: perfluorodecanoic acid; PFDoDA: perfluorododecanoic acid; PFHpA: perfluoroheptanoic acid; PFHpS: perfluoroheptanesulfonic scid; PFHxS: perfluorohexanesulfonic acid; PFNA: perfluorononanoic acid; PFOA: perfluorooctanoic acid; PFOS: perfluorooctane sulfonate; PFOSA: perfluorooctanesulfonamide; PFPA: perfluoroalkyl phosphonic acid; PFUA: perfluoroundecanoic acid; PFUnDA: perfluoroundecanoic acid.

a.: The concentration unit of unmarked pollutants is ng/mL.

**Supplementary Table 4** The correlation between other PCPs exposure and the risk of AD

| **Pollutant** | **Concentration^a.^** | **Method** | **Country/Region** | **Population** | **Sample** | **Sample size** | **Correlation** | **Risk indicator;**  **95%CI** | **P-value** | **reference** |
| --- | --- | --- | --- | --- | --- | --- | --- | --- | --- | --- |
| TCS | 1050 (GM) | cohort study | Taiwan | 3 Y | urine (3 Y) | 453 | + | O 1.22; 1.05-1.41 | < 0.05 | (29) |
|  |  |  |  | 6 Y |  |  | - | 1.00; 0.83-1.21 | ＞0.05 |  |
|  | 370 |  |  | 6 Y | urine (6 Y) | 200 | + | 1.26; 1.02-1.55 | < 0.05 |  |
| TCS | <LoD (nmol/mL) | cross-sectional study | Tokyo | 0-15 Y | urine | 138 | - | O 0.268; 0.055-1.310 | / | (30) |
| TCS | 0.26 (GM; μg/g Cr) | cross-sectional study | Korea | 3-5 Y | urine | 556 | - | O 0.98; 0.52-1.86 | <0.05 | (31) |
|  | 0.25 |  |  | 6-11 Y |  | 701 | + | 1.27; 1.08-1.50 | / |  |
|  | 0.12 |  |  | 12-17 Y |  | 731 | - | 1.13; 0.91-1.40 | / |  |
| phenol | 3.63 (Mean; ng/m^3^/year) | cohort study | Minsk | 0-2 Y | environment | 1965 | + | O 1.724; 1.091-2.723 | 0.020 | (32) |
| BPAG | 3.36/3.09 (GM; μg/g Cr)^b.^ | longitudinal study | Seoul | 3-7 Y (♂) | urine | 18 (460 times) | - | O 1.79; 0.91-3.52 | / | (33) |
| BPA | 0.00079 (GM) | prospective cohort study | Korea | 6 M | urine (early intrauterine period) | 413 | - | Rd 0.026; (−0.128)-0.179 | / | (34) |
|  | 0.00119 |  |  |  | urine (late intrauterine period) |  | - | 0.082; (−0.060)-0.224 | / |  |
| BPA | 0.00241 (GM) | cross-sectional study | Korea | 3-5 Y | urine | 571 | - | odds 1.21; 0.73-2.02 | / | (17) |
| BPS | 0.000028 |  |  |  |  |  | - | 1.10; 0.85-1.43 | / |  |
| BPF | 0.000091 |  |  |  |  |  | - | 0.80; 0.47-1.36 | / |  |
| BPA | 0.00170 |  |  | 6-11 Y |  | 887 | + | 1.31; 1.06-1.61 | / |  |
| BPS | 0.000036 |  |  |  |  |  | - | 1.15; 0.94-1.40 | / |  |
| BPF | 0.000110 |  |  |  |  |  | - | 1.19; 0.98-1.44 | / |  |
| BPA | 0.00096 (Me) | cohort study | USA | 0-3 Y | urine (mother) | 628 | - | O 1.08; 0.85-1.39 | / | (35) |
| BPF | 0.00044 |  |  |  |  | 165 | - | 1.11; 0.65-1.89 | / |  |
| BPS | 0.00029 |  |  |  |  | 599 | - | 0.84; 0.67-1.05 | / |  |
| Benzylmercapturic acid | 0.25 (LoD) | cross-sectional study | Korea | 7-8 Y | urine | 149 | + | O 3.52; 1.34-8.94 | / | (36) |
| *p*-methyl-benzoic acid | 32.2 (Me; μg/g Cr) |  |  |  |  |  | - | 1.64; 0.79-3.41 | / |  |
| *o-*methyl-benzoic acid | 236.4 |  |  |  |  |  | - | 0.72; 0.35-1.51 | / |  |
| tt, cc-Muconic acid | 14 (LoD) |  |  |  |  |  | - | 1.52; 0.72-3.21 | / |  |
| Phenylglyoxylic acid | 290 |  |  |  |  |  | - | 1.29; 0.62-2.67 | / |  |
| Thiazolidine-4-carboxylic acid | 84 |  |  |  |  |  | - | 0.78; 0.38-1.62 | / |  |
| Mandelic acid^c.^ | 168 |  |  |  |  |  | + | 3.77; 1.19-11.92 | / |  |
| Benzene | 0.5 (GM; ppb) | prospective longitudinal study | Seou | 2-7 Y | environment | 30 | - | CoR 12.00; (-0.93)-26.62 | 0.070 | (37) |
| Toluene | 42.5 |  |  |  |  |  | - | 7.78; (-1.19)-17.57 | 0.091 |  |
| Ethyl-benzene | 1.8 |  |  |  |  |  | - | 4.29; (-5.08)-14.58 | 0.382 |  |
| Xylene | 2.8 |  |  |  |  |  | - | 2.68; (-1.83)-7.40 | 0.249 |  |
| Styrene | 0.7 |  |  |  |  |  | - | 2.95; (-4.94)-11.49 | 0.475 |  |
| VOC | 80.5 |  |  |  |  |  | - | 0.03; (-0.25)-0.31 | 0.843 |  |
| VOC | 412.4; 455.8 (Mean; ppb)^d.^ | longitudinal study | Nowon-guKorea | ≥20 Y | environment | 25 | + | O 1.05^e.^ | ＜0.05 | (38) |
|  |  |  |  | 0-15 Y |  | 25 | - | 1.03 | ＞0.05 |  |
| VOC | 242.1 (the third quartile; μg/m^3^) | prospective cohort study | Korea | 3 Y | environment | 105 | + | O 3.116; 1.041-9.323 | / | (39) |
| VOC | / | cohort study | Ulsan | local | urine | 1225 | + | O 1.549; 1.013-2.370 | / | (40) |

Y: years old; H: Hazard Ratio; O: Odds Ratio; R: Relative Risk; Rd: Risk difference; +: Increased risk of AD; -: not related to increased risk of AD onset; CoR: % change of risk; GM: geometric mean; LoD: limit of detection; Me: median; GM: geometric mean; LoD: limit of detection; BPAG: bisphenol A glucuronide; BPS: bisphenol S; BPF: bisphenol F.

a.: The concentration unit of unmarked pollutants is μg/mL; b.: The first number is for the concentration of pollutants in the morning, and the last number is for the concentration of pollutants in the afternoon. c.:For this pollutant, the author provides a comparison between the fourth quartile and the first quartile; d.: The first number is for energy-efficient homes, and the last number is for conventional apartment; e.: The author used images to represent a 95% confidence interval, but did not specify the exact number.

**References**

1. El-Heis S, Crozier SR, Harvey NC, Healy E, Godfrey KM. Early life exposure to antibiotics and laxatives in relation to infantile atopic eczema. Pediatr Allergy Immunol. (2023) 34(5):e13964.

2. Mitre E, Susi A, Kropp LE, Schwartz DJ, Gorman GH, Nylund CM. Association Between Use of Acid-Suppressive Medications and Antibiotics During Infancy and Allergic Diseases in Early Childhood. JAMA PEDIATR. (2018) 172(6):e180315.

3. Noh Y, Jeong HE, Choi A, Choi EY, Pasternak B, Nordeng H, et al. Prenatal and Infant Exposure to Acid-Suppressive Medications and Risk of Allergic Diseases in Children. JAMA PEDIATR. (2023) 177(3):267-77.

4. Mulder B, Schuiling-Veninga CC, Bos HJ, De Vries TW, Jick SS, Hak E. Prenatal exposure to acid-suppressive drugs and the risk of allergic diseases in the offspring: a cohort study. CLIN EXP ALLERGY. (2014) 44(2):261-9.

5. Lin HC, Chen YY, Lin HL, Uang YS, Ho Y, Wang LH. Association between acid-suppressive drug use and atopic dermatitis in patients with upper gastrointestinal diseases: A population-based retrospective cohort study. J CLIN PHARM THER. (2021) 46(3):786-93.

6. Tsuchida T, Yoshida S, Takeuchi M, Kawakami K. Large-scale health insurance study showed that antibiotic use in infancy was associated with an increase in atopic dermatitis. ACTA PAEDIATR. (2022) 111(3):607-13.

7. Kohno C, Kaneko K, Takahashi K, Ohya Y, Nakajima H, Murashima A. Intrauterine exposure to immunosuppressants influences the development of postnatal allergic diseases. ALLERGY. (2021) 76(9):2890-3.

8. Li CY, Dai YX, Chang YT, Bai YM, Tsai SJ, Chen TJ, et al. Prenatal exposure to acetaminophen increases the risk of atopic dermatitis in children: A nationwide nested case-control study in Taiwan. Pediatr Allergy Immunol. (2021) 32(5):1080-8.

9. Rutter CE, Silverwood RJ, Williams HC, Ellwood P, Asher I, Garcia-Marcos L, et al. Are Environmental Factors for Atopic Eczema in ISAAC Phase Three due to Reverse Causation? J INVEST DERMATOL. (2019) 139(5):1023-36.

10. Chang YC, Wu MC, Wu HJ, Liao PL, Wei JC. Prenatal and early-life antibiotic exposure and the risk of atopic dermatitis in children: A nationwide population-based cohort study. Pediatr Allergy Immunol. (2023) 34(5):e13959.

11. Ahmetaj L, Torres AEM, Ahmetaj Y, Gashi V, Kurhasani X, Berisha VL, et al. Prevalence of atopic eczema in adolescents from a very low prevalence area (Kosovo): role of wheezing, gender, exercise, and paracetamol. ALLERGOL IMMUNOPATH. (2024) 52(6):40-50.

12. Al NS, Abouammoh N, Althagafi W, Alomary SA, Almutairi AS, Assiri AM, et al. Prevalence, severity, and risk factors of eczema among young children and adolescents in Saudi Arabia: A national cross-sectional study, 2019. J Allergy Clin Immunol Glob. (2024) 3(4):100299.

13. Kuo HC, Yang YL, Ho SC, Guo MM, Jiang JH, Huang YH. General anesthesia exposure in early life reduces the risk of allergic diseases: A nationwide population-based cohort study. Medicine (Baltimore). (2016) 95(28):e4269.

14. Kim DC, Choi YW, Lee ES, Choi JW. No Association Between First Exposure to General Anaesthesia and Atopic Dermatitis in the Paediatric Population. Acta Derm Venereol. (2022) 102:v813.

15. Haerskjold A, Stokholm L, Linder M, Thomsen SF, Bergman G, Berglind IA, et al. Palivizumab Exposure and the Risk of Atopic Dermatitis, Asthma and Allergic Rhinoconjunctivitis: A Cross-National, Population-Based Cohort Study. Paediatr Drugs. (2017) 19(2):155-64.

16. Choi WJ, Kwon HJ, Hong S, Lim WR, Kim H, Kim J, et al. Potential nonmonotonous association between di(2-ethylhexyl) phthalate exposure and atopic dermatitis in Korean children. Br J Dermatol. (2014) 171(4):854-60.

17. Hwang M, Choi K, Park C. Urinary levels of phthalate, bisphenol, and paraben and allergic outcomes in children: Korean National Environmental Health Survey 2015-2017. SCI TOTAL ENVIRON. (2022) 818:151703.

18. Ait BY, Araki A, Kawai T, Tsuboi T, Saito I, Yoshioka E, et al. Exposure to phthalates in house dust and associated allergies in children aged 6-12years. ENVIRON INT. (2016) 96:16-23.

19. Stelmach I, Majak P, Jerzynska J, Podlecka D, Stelmach W, Polańska K, et al. The effect of prenatal exposure to phthalates on food allergy and early eczema in inner-city children. ALLERGY ASTHMA PROC. (2015) 36(4):72-8.

20. Kampouri M, Gustin K, Stråvik M, Barman M, Sandin A, Sandberg AS, et al. Associations of gestational and early-life exposure to toxic metals and fluoride with a diagnosis of food allergy or atopic eczema at 1 year of age. ENVIRON INT. (2023) 178:108071.

21. Sevelsted A, Pedersen CT, Gürdeniz G, Rasmussen MA, Schullehner J, Sdougkou K, et al. Exposures to perfluoroalkyl substances and asthma phenotypes in childhood: an investigation of the COPSAC2010 cohort. EBIOMEDICINE. (2023) 94:104699.

22. Kvalem HE, Nygaard UC, Lødrup CK, Carlsen KH, Haug LS, Granum B. Perfluoroalkyl substances, airways infections, allergy and asthma related health outcomes - implications of gender, exposure period and study design. ENVIRON INT. (2020) 134:105259.

23. Wen HJ, Wang SL, Chen PC, Guo YL. Prenatal perfluorooctanoic acid exposure and glutathione s-transferase T1/M1 genotypes and their association with atopic dermatitis at 2 years of age. PLOS ONE. (2019) 14(1):e210708.

24. Impinen A, Longnecker MP, Nygaard UC, London SJ, Ferguson KK, Haug LS, et al. Maternal levels of perfluoroalkyl substances (PFASs) during pregnancy and childhood allergy and asthma related outcomes and infections in the Norwegian Mother and Child (MoBa) cohort. ENVIRON INT. (2019) 124:462-72.

25. Chen Q, Huang R, Hua L, Guo Y, Huang L, Zhao Y, et al. Prenatal exposure to perfluoroalkyl and polyfluoroalkyl substances and childhood atopic dermatitis: a prospective birth cohort study. Environ Health. (2018) 17(1):8.

26. Rudzanova B, Vlaanderen J, Kalina J, Piler P, Zvonar M, Klanova J, et al. Impact of PFAS exposure on prevalence of immune-mediated diseases in adults in the Czech Republic. ENVIRON RES. (2023) 229:115969.

27. Wen HJ, Wang SL, Chuang YC, Chen PC, Guo YL. Prenatal perfluorooctanoic acid exposure is associated with early onset atopic dermatitis in 5-year-old children. CHEMOSPHERE. (2019) 231:25-31.

28. Impinen A, Nygaard UC, Lødrup CK, Mowinckel P, Carlsen KH, Haug LS, et al. Prenatal exposure to perfluoralkyl substances (PFASs) associated with respiratory tract infections but not allergy- and asthma-related health outcomes in childhood. ENVIRON RES. (2018) 160:518-23.

29. Lin MH, Chiu SY, Ho WC, Chi KH, Liu TY, Wang IJ. Effect of triclosan on the pathogenesis of allergic diseases among children. J Expo Sci Environ Epidemiol. (2022) 32(1):60-8.

30. Mitsui-Iwama M, Yamamoto-Hanada K, Fukutomi Y, Hirota R, Muto G, Nakamura T, et al. Exposure to paraben and triclosan and allergic diseases in Tokyo: A pilot cross-sectional study. Asia Pac Allergy. (2019) 9(1):e5.

31. Choi YH, Huh DA, Moon KW. Exposure to biocides and its association with atopic dermatitis among children and adolescents: A population-based cross-sectional study in South Korea. Ecotoxicol Environ Saf. (2024) 270:115926.

32. Belugina IN, Yagovdik NZ, Belugina OS, Belugin SN. Outdoor environment, ozone, radionuclide-associated aerosols and incidences of infantile eczema in Minsk, Belarus. J Eur Acad Dermatol Venereol. (2018) 32(11):1977-85.

33. Kim EH, Jeon BH, Kim J, Kim YM, Han Y, Ahn K, et al. Exposure to phthalates and bisphenol A are associated with atopic dermatitis symptoms in children: a time-series analysis. Environ Health. (2017) 16(1):24.

34. Lee S, Park SK, Park H, Lee W, Lee JH, Hong YC, et al. Joint association of prenatal bisphenol-A and phthalates exposure with risk of atopic dermatitis in 6-month-old infants. SCI TOTAL ENVIRON. (2021) 789:147953.

35. Miller RL, Wang Y, Aalborg J, Alshawabkeh AN, Bennett DH, Breton CV, et al. Prenatal exposure to environmental bisphenols over time and their association with childhood asthma, allergic rhinitis and atopic dermatitis in the ECHO consortium. Environmental pollution (Barking, Essex : 1987). (2024) 366:125415.

36. Ha EK, Kim JH, Park D, Lee E, Lee SW, Jee HM, et al. Personal Exposure to Total VOC Is Associated With Symptoms of Atopic Dermatitis in Schoolchildren. J KOREAN MED SCI. (2022) 37(8):e63.

37. Kim EH, Kim S, Lee JH, Kim J, Han Y, Kim YM, et al. Indoor air pollution aggravates symptoms of atopic dermatitis in children. PLOS ONE. (2015) 10(3):e119501.

38. Lim AY, Yoon M, Kim EH, Kim HA, Lee MJ, Cheong HK. Effects of mechanical ventilation on indoor air quality and occupant health status in energy-efficient homes: A longitudinal field study. SCI TOTAL ENVIRON. (2021) 785:147324.

39. Kwon JH, Kim E, Chang MH, Park EA, Hong YC, Ha M, et al. Indoor total volatile organic compounds exposure at 6 months followed by atopic dermatitis at 3 years in children. Pediatr Allergy Immunol. (2015) 26(4):352-8.

40. Kim AR, Bang JH, Lee S, Sim CS, Kim Y, Lee J. Distribution of volatile organic compounds by distance from industrial complexes and potential health impact on the residents. Int J Environ Health Res. (2024) 34(12):4202-13.
